# Effect of a giant meteorite impact on Paleoarchean surface environments and life

**DOI:** 10.1073/pnas.2408721121

**Published:** 2024-10-21

**Authors:** Nadja Drabon, Andrew H. Knoll, Donald R. Lowe, Stefano M. Bernasconi, Alec R. Brenner, David A. Mucciarone

**Affiliations:** ^a^Department of Earth and Planetary Sciences, Harvard University, Cambridge, MA 02138; ^b^Department of Earth and Planetary Sciences, Stanford University, Stanford, CA 94305; ^c^Department of Earth Sciences, ETH Zürich, Zürich 8092, Switzerland; ^d^Oceans Department, Stanford University, Stanford, CA 94305

**Keywords:** Archean, early Earth, meteorite impact, habitability

## Abstract

Large meteorite impacts must have strongly affected the habitability of the early Earth. Rocks of the Archean Eon record at least 16 major impact events, involving bolides larger than 10 km in diameter. These impacts probably had severe, albeit temporary, consequences for surface environments. However, their effect on early life is not well understood. Here, we analyze the sedimentology, petrography, and carbon isotope geochemistry of sedimentary rocks across the S2 impact event (37 to 58 km carbonaceous chondrite) forming part of the 3.26 Ga Fig Tree Group, South Africa, to evaluate its environmental effects and biological consequences. The impact initiated 1) a giant tsunami that mixed Fe^2+^-rich deep waters into the Fe^2+^-poor shallow waters and washed debris into coastal areas, 2) heating that caused partial evaporation of surface ocean waters and likely a short-term increase in weathering and erosion on land, and 3) injection of P from vaporization of the S2 bolide. Strata immediately above the S2 impact event contain abundant siderites, which are associated with organic matter and exhibit light and variable δ^13^C_carb_ values. This is consistent with microbial iron cycling in the wake of the impact event. Thus, the S2 impact likely had regional, if not global, positive and negative effects on life. The tsunami, atmospheric heating, and darkness would likely have decimated phototrophic microbes in the shallow water column. However, the biosphere likely recovered rapidly, and, in the medium term, the increase in nutrients and iron likely facilitated microbial blooms, especially of iron-cycling microbes.

## Significance

Giant meteorite impacts during Earth’s early history likely had significant effects on early life. We studied the effects on the surface environment and life of a Paleoarchean impactor ~50 to 200× larger than the famous K-Pg impactor. The impact caused a tsunami, partial ocean evaporation, and darkness that likely harmed shallow-water photosynthetic microbes in the short-term, while life in the deeper oceans and hyperthermophiles were less impacted. The impact also released phosphorus into the environment, and the tsunami brought iron-rich deep-water to the surface. As a consequence, there was a temporary bloom of iron-cycling microbes. Giant impacts were not just agents of destruction but also conferred transient benefits on early life.

Numerous studies have shown that large impacts have severe consequences for the surface environment and, thus, can potentially severely affect life (1–5). Most famously, the impact of the 10 km Chicxulub bolide at the K-Pg boundary profoundly disrupted the global environment, including the initiation of a tsunami (6), a sharp, if transient, drop in surface temperatures (7) followed by moderate, longer-term warming (8), short-term darkness (9), and ocean acidification (10). As a result, some 40% of animal genera and as many as 60 to 80% of animal species became extinct (3, 11). Carbon isotopes reveal a disturbance in the ocean’s biological pump due to a severe drop to marine productivity (12, 13). On the other hand, in the longer-term, bolide-induced environmental disruption created new opportunities for survivors, e.g., the mammalian radiation that followed closely on the heels of dinosaur extinction (14). Moreover, and possibly more relevant to an early microbial world, it has been proposed that bolide and target rock vaporization may inject significant volumes of biologically relevant sulfur, phosphorus, and iron into the biosphere (15) for subsequent use by microbial communities.

On the Archean Earth, the impact flux was substantially higher than today; it has been estimated that giant impactors (>10 km in diameter) pummeled the Earth at least every 15 Ma (16). These events likely had severe effects on Earth’s nascent biosphere, but their specific influence is still poorly understood. Modeling predicts that impactors >440 km in diameter could have annihilated much of the biosphere due to evaporation of the entire ocean; impactors ~190 km in diameter would have evaporated the preexisting photic zone (4). Geological evidence supports the partial evaporation (10 s of meters) of oceans by impacts in the form of silica crusts above two Paleoarchean impact layers (17). Mass mortality of non-hyperthermophile microbes in shallow waters was inferred. Early Earth impacts would also have generated large clouds of dust that may have blocked sunlight (9). In addition, essentially all Archean impact deposits show evidence for tsunamis, initiated by oceanic impacts or by slope failure caused by impact-generated seismic activity (17–20). Seismic waves released from the impacts may additionally have caused fracturing of the upper crust (21). On the other hand, impacts may have also conferred benefits on the biosphere; for example, some work has hypothesized that impact-initiated tsunamis mixed nutrient-rich deep waters to the upper water column, making these nutrients available to local ecosystems (5, 19).

The effect of a giant impact on life depends on the size and type of the impactor, the target material, the conditions of the atmosphere and hydrosphere, and the type of life present at the time of impact. While progress has been made on the environmental effects of some Archean impacts, little is known about their effects on early life. To address this issue, we studied two sections across the 3.26 Ga S2 impact event recorded in the lowermost Fig Tree Group, South Africa (informally named “Umbaumba” and “Bruce’s Hill”; *SI Appendix*, Fig. S1). The S2 impactor had an estimated bolide diameter of 37 to 58 km (22), by mass ~50 to 200 times larger than the K-Pg bolide. We analyzed the sedimentology, petrography, trace element geochemistry, total organic carbon (TOC) content, and carbon isotope geochemistry of carbonaceous matter and carbonate, with the goal of evaluating the effects of the S2 impact on early surface environments and life.

## Geological Background

Eight impact events (termed S1 through S8) have been identified throughout the stratigraphy of the 3.6 to 3.2 Ga Barberton Greenstone Belt (17, 19), located in the Kaapvaal Craton of southern Africa (*SI Appendix*, Fig. S1). The impacts are identified largely by the presence of mm-scale spherules together with Ir anomalies (19, 23, 24) and extraterrestrial Cr isotope signatures (25). The S2 spherule bed marks the base of the Fig Tree Group in at least six different structural belts (*SI Appendix*, Fig. S1*C*). The impact occurred at ~3.26 Ga based on an immediately overlying 3,258 ± 3 Ma tuff (26). The rocks are well preserved, as this area experienced only lower greenschist facies metamorphism (27, 28) with limited to no shearing. Furthermore, seafloor sediments were silicified soon after deposition (29–31), preserving primary textural, environmental, and biogeochemical information with excellent fidelity.

The S2 spherule bed is underlain by the Mendon Formation, which consists of komatiitic volcanic rocks with thin, interbedded chert layers, representing silicified sediments and primary chemical precipitates. The sediments were deposited in a marine environment that ranged in water depth from below storm wave base to relatively shallow water (31). The dominant lithologies are black-and-white banded chert (BWBC), banded ferruginous chert, and black chert. All chert types are composed predominantly of microcrystalline quartz with either minimal impurities (white chert), variable amounts of carbonaceous material (black chert), or siderite, hematite, and magnetite (ferruginous chert). The basal Fig Tree Group above S2 is often characterized by banded ferruginous chert that transitions upward into mudstones, banded iron formation, and turbiditic sandstones (32–34). Fe-rich cherts occur predominantly in deeper-water facies, below storm wave base, and are largely absent in shallow-water sections. This suggests Fe^2+^-enriched deep-water and Fe^2+^-depleted shallow water during this time (30, 31, 35, 36).

Carbonaceous material is abundant in both sections (31), including both simple to complex carbonaceous grains and rare fine carbonaceous laminations. Identifying the origin of this carbonaceous matter is difficult due to its lack of microfossils, biomarkers, and/or sedimentary reworking. However, the fine carbonaceous laminations resemble microbial mats, an interpretation supported by rare roll-up structures and three-dimensional (3D) lamina networks (*SI Appendix*, Figs. S4 and S5). Many of the simple to complex carbonaceous grains may represent the remains of planktonic microbes and/or ripped-up microbial mats (29, 31). Importantly, an abiogenic hydrothermal origin through Fischer Tropsch-type synthesis is unlikely due to the abundance of carbonaceous matter and the lack of identifiable hydrothermal vents or polymetallic deposits (31).

## Results

### Sedimentology and Petrography.

**Fig. 1.**
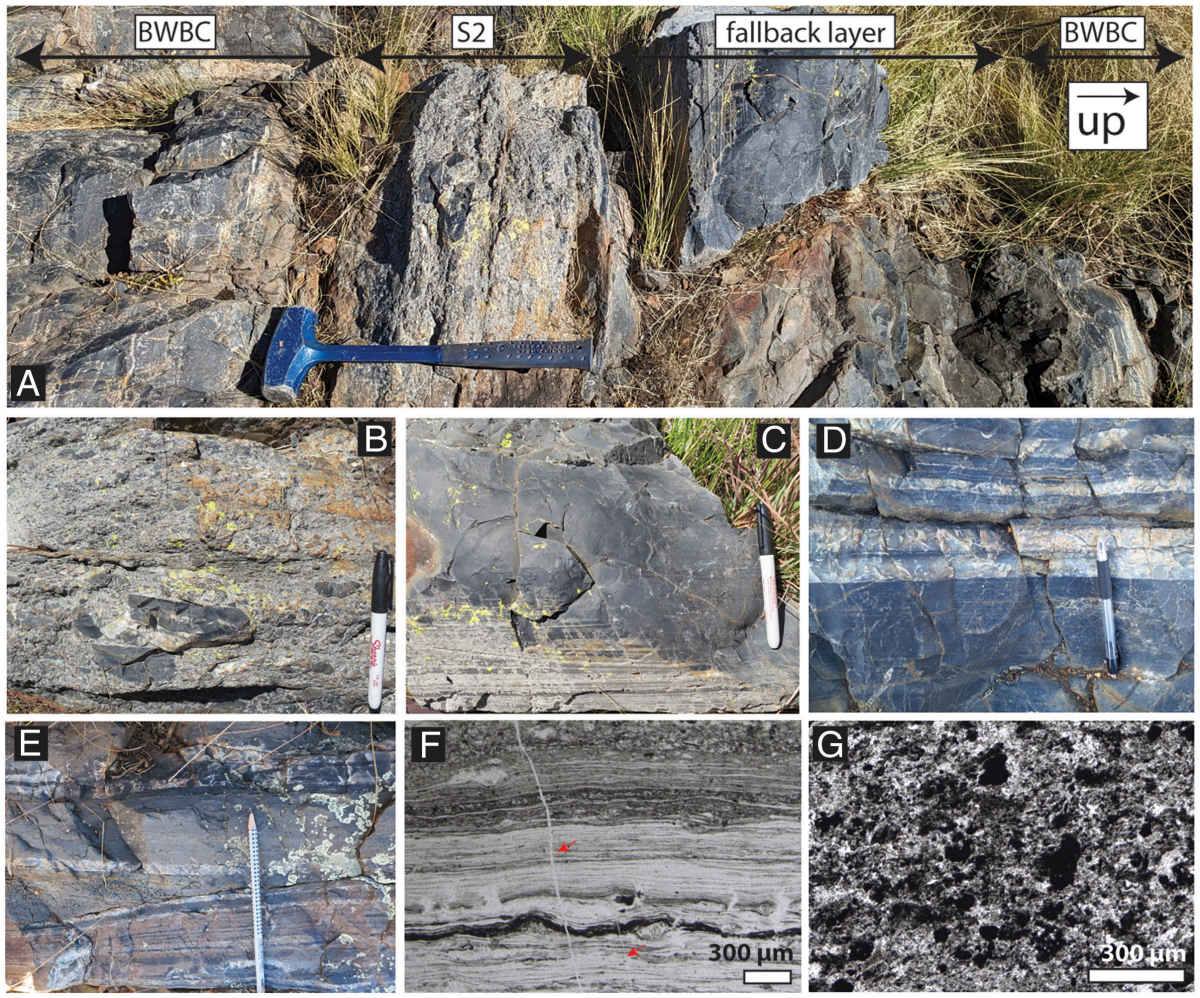
Rock and thin section images of the Bruce’s Hill and Umbaumba sections. (*A*–*C*) Outcrop photos of the Umbaumba section. (*A*) Overview of the Umbaumba section showing, from base to top, BWBC, S2, fallback layer, and BWBC. (*B*) S2 spherule bed. (*C*) Lower part of the fallback layer showing fine laminations. This black chert is composed of silicified carbonaceous matter, siliciclastic debris, and impact-generated dust settling out of the atmosphere. (*D* and *E*) Outcrop photos of the Bruce’s Hill section. (*D*) BWBC below S2. (*E*) Alternating siliciclastic and siderite-rich chert beds. (*F*–*G*) Representative thin section images of carbonaceous matter. (*F*) Laminated carbonaceous chert below S2 in the Umbaumba section (*SI Appendix*, Fig. S4). Red arrows indicate fractures filled by chert. (*G*) Clots of carbonaceous matter and other siliciclastic debris from the fallback later in the Umbaumba section.

**Fig. 2.**
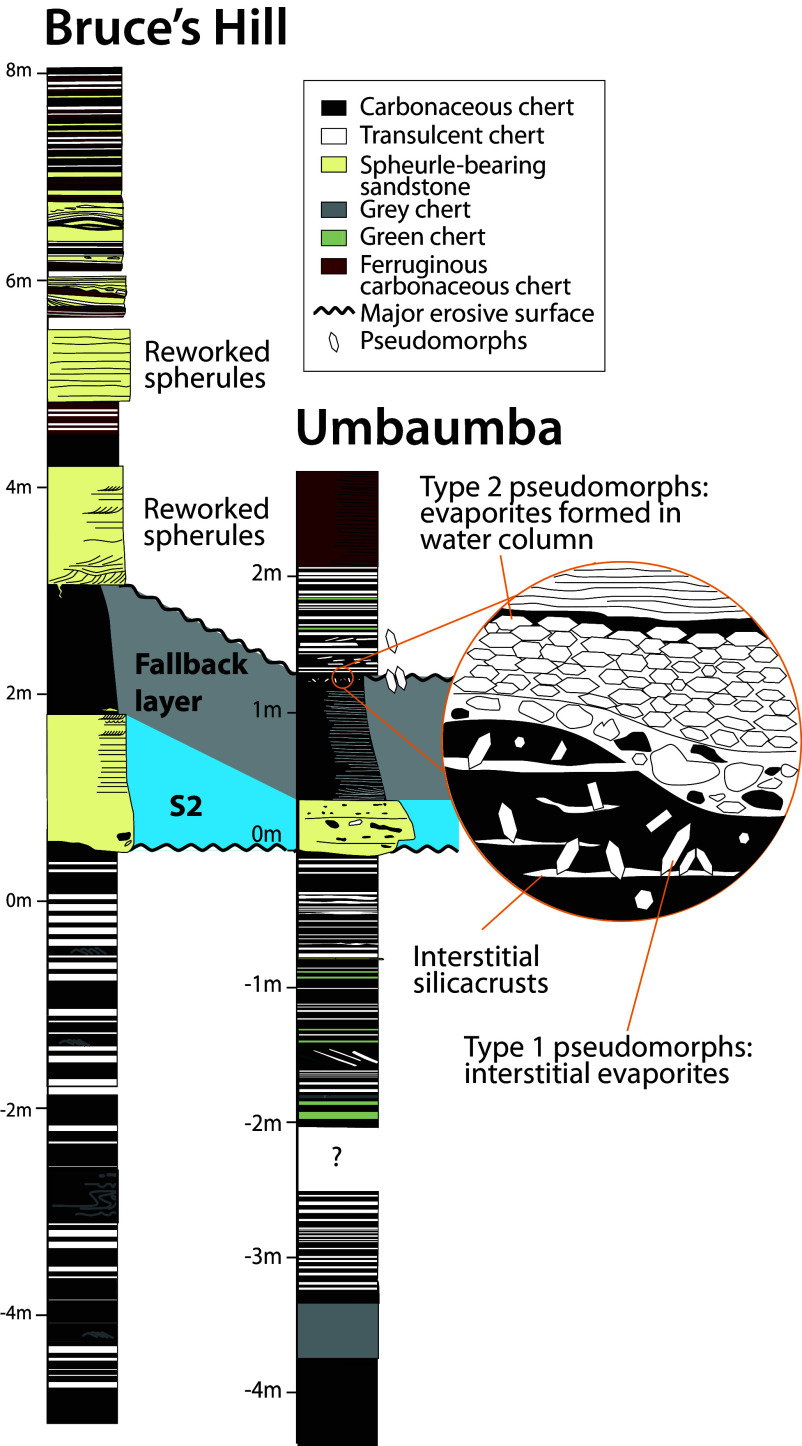
Stratigraphic sections of the Bruce’s Hill and Umbaumba locations. The *Inset* shows the top of the fallback layer.

While different in detail, the sedimentology and petrography of the two sections (Figs. 1 and 2) show similar transitions across the S2 impact event:

#### Before S2.

Background sedimentation in both sections prior to the S2 impact was dominated by BWBC, black chert, and minor silicified volcanic ash (Fig. 1 *A*, *D*, and *F* and *SI Appendix*, Figs. S2 and S3). In the Umbaumba section, black chert bands consist of fine carbonaceous laminations, simple and complex carbonaceous grains, and silica granules (Fig. 1*F* and *SI Appendix*, Figs. S3–S6). There is little admixed siliciclastic debris other than chert intraclasts. Sedimentary structures are dominated by planar lamination (Fig. 1*F* and *SI Appendix*, Fig. S2). Occasional chert plate conglomerates consist of platy white chert clasts in a matrix of black chert (Fig. 2 and *SI Appendix*, Fig. S2), suggesting that the white but not the back chert had lithified at the time of erosion. Graded beds are restricted largely to ash fall layers. In the Bruce’s Hill section, black chert bands contain simple carbonaceous grains, silica granules, and siliciclastic detritus. Complex carbonaceous grains and carbonaceous laminations are rare. Sedimentary structures are dominated by fine lamination, cross-laminations, and soft-sediment folding (Figs. 1*D* and 2 and *SI Appendix*, Fig. S3).

#### S2 spherule beds.

The S2 spherule bed in both sections is composed of spherules admixed with poorly sorted, cobble- to fine sand-sized debris (Figs. 1*B* and 2 and *SI Appendix*, Figs. S2 and S3). Large clasts are composed of reworked black and translucent chert eroded from the sea floor. The base of the S2 spherule bed is erosive in both sections. In the Umbaumba section, the spherule bed is ~20 cm thick and normally graded; it contains ~5% spherules (Figs. 1*B* and 2 and *SI Appendix*, Fig. S2). In the Bruce’s Hill section, three separate sandstone beds (120, 100, and 70 cm thick, Fig. 2) are separated by silicified, fine-grained, Fe-rich materials (fallback layers). The sandstone beds show an upward fining trend from conglomeratic to medium-grained sandstone. The beds are massive at the base to flat- and cross-laminated at the top, indicating a decline in current energy. The second sandstone shows a sand injectite into the underlying fallback layer (*SI Appendix*, Fig. S7). The third spherule bed shows the highest abundance of spherules (~10%).

#### Fallback layers.

In both sections, the spherule bed is overlain by ~1 m of black, fine-grained silicified sediment. The beds are normally graded from medium- to very fine-grained sandstone (Fig. 1 *A* and *C* and 2 and *SI Appendix*, Fig. S8). Thin lenses of coarser material and cross-lamination at their bases suggest some weak current activity as deposition began. Above, they are structureless to finely laminated, indicating that they may have settled out of suspension. The silicified sediment is composed mainly of carbonaceous material, mica, and fine siliciclastic debris (Fig. 1*G*). While in the Umbaumba section, only one such fallback layer is present, both the first and second spherule-bearing sandstone beds in the Bruce’s Hill section are overlain by fallback layers, with the second fallback layer transitioning into ~35 cm of banded ferruginous chert and jasper (Fig. 2). Neither fallback layer is fully preserved, as they show erosive contact with overlying sandstone beds (Fig. 2 and *SI Appendix*, Fig. S3).

#### Pseudomorphs.

**Fig. 3.**
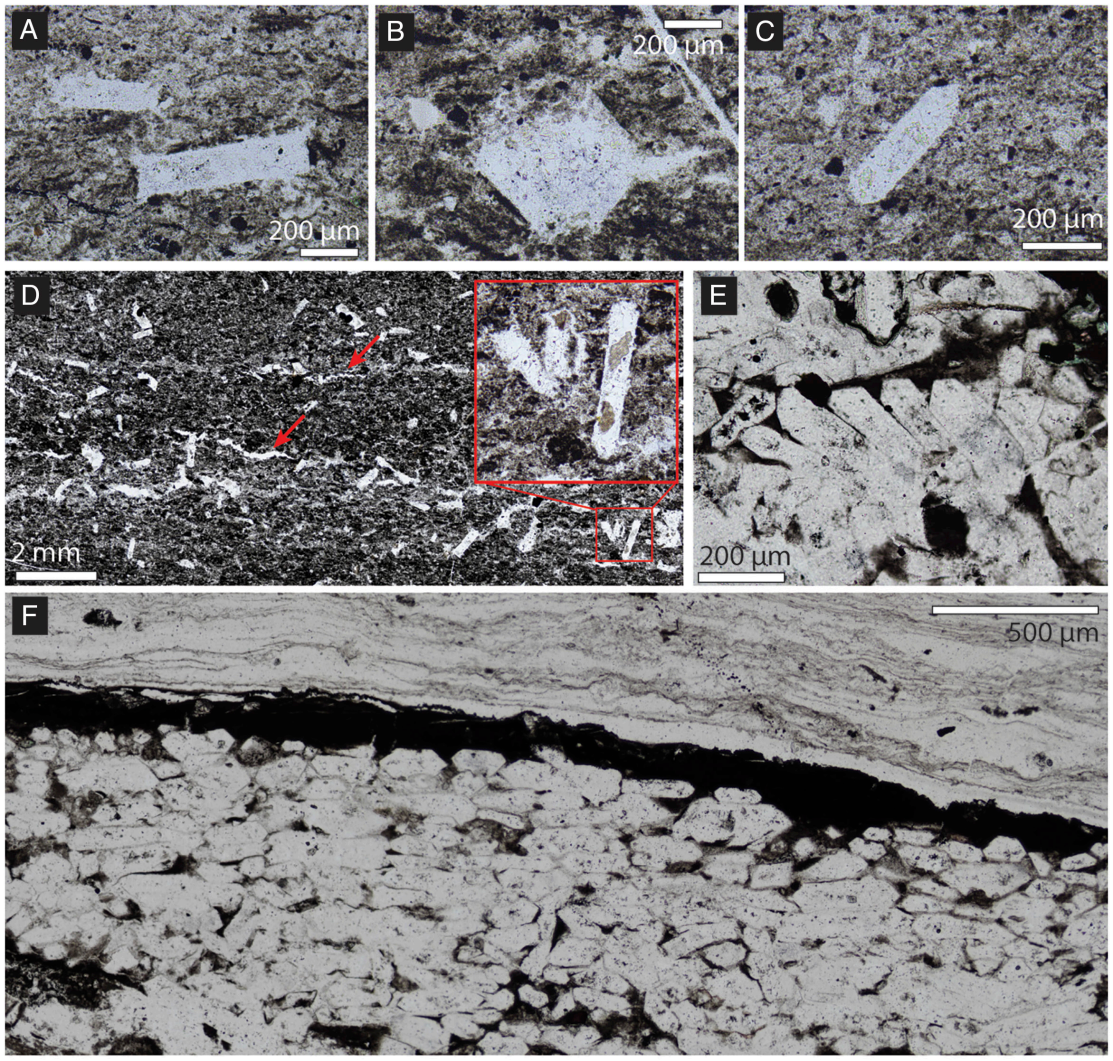
Photomicrographs of pseudomorphs. (*A*–*C*) Representative Type 1 pseudomorphs from the fallback layer. (*D*) Randomly oriented Type 1 pseudomorphs and thin, discontinuous silica crusts (red arrows). (*E*) Type 2 pseudomorphs imbricated from slight sedimentary reworking. (*F*) Tightly packed Type 2 pseudomorphs showing preferred orientation parallel to bedding and minor organic matter. The pseudomorphs are overlain by a layer of organic matter. See *SI Appendix,* Figs. S9 and S10 for crystal measurements and more images.

In the Umbaumba section, pseudohexagonal, idiomorphic crystal pseudomorphs occur in the upper fallback layer and above (Figs. 2 and 3 and *SI Appendix*, Figs. S9 and S10). The pseudomorphs are now composed of silica, and, in some samples, pyrite (*SI Appendix*, Fig. S10). This composition is similar to chert fracture fills within the same samples and speaks to a replacement texture. There are two types of pseudomorphs: those oriented randomly (Type 1, Fig. 3 *A*–*D* and *SI Appendix*, Fig. S9) and those oriented horizontally (Type 2, Fig. 3 *E* and *F* and *SI Appendix*, Fig. S9). Type 1 pseudomorphs occur within the upper fallback layer, and up to 30 cm above in the banded chert layer (Fig. 2). These randomly oriented pseudomorphs are up to 1,000 × 400 µm in size, thus more than a magnitude larger than the surrounding sediment (*SI Appendix*, Fig. S9). In general, they increase in size and abundance toward the top of the fallback layer, while the clastic particle size decreases (*SI Appendix*, Fig. S8). The pseudomorphs are associated with bedding-parallel crusts of silica (Figs. 2 and 3*D*). Type 2 pseudomorphs only occur immediately above an erosional unconformity truncating the top of the fallback layer (Fig. 2). Here, Type 2 pseudomorphs form a ~1 cm thick layer composed of siliciclastic debris that contains an increasing amount of pseudomorphs toward the top. Within this layer, some pseudomorphs show imbrication (Fig. 3*E*). At the top, the bed is composed of tightly packed pseudomorphs. Type 2 pseudomorphs are oriented predominantly parallel to subparallel to bedding (Figs. 3 *E* and *F* and *SI Appendix*, Figs. S9 and S10) and are slightly smaller than Type 1 pseudomorphs, up to 700 ×150 µm (*SI Appendix*, Fig. S9).

#### After S2.

Above the fallback layers, both sections show significant changes in sedimentology and petrography. Most characteristically, both show an increase in siliciclastic detritus and Fe-rich minerals. In the Umbaumba section, the fallback layer is followed by 70 cm of BWBC that contains abundant siliciclastic sand and chert plates (Fig. 2). This interval contains randomly oriented Type 1 pseudomorphs. This unit is immediately followed by an 80-cm-thick unit of laminated to cross-laminated ferruginous chert (Fig. 2). This rock includes alternating laminations dominated by either siderite (~20 to 30 µm rhombs) or pyrite (~10 to 100 µm euhedra). It is noteworthy that siderite appears to cluster around organic carbon particles in many places (*SI Appendix*, Fig. S12).

**Fig. 4.**
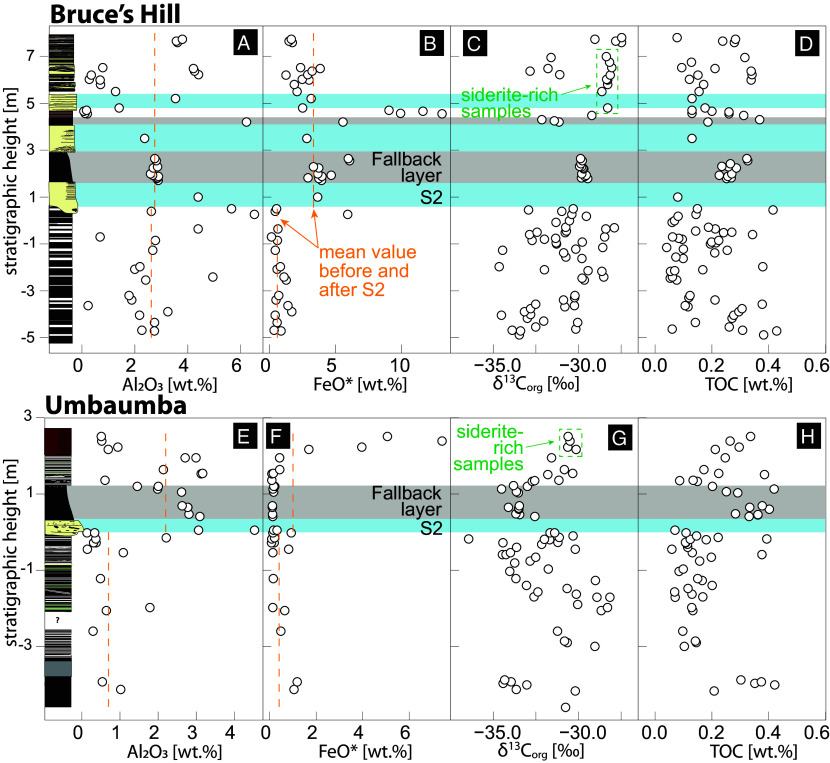
Geochemical variations across the S2 impact event. Bruce's Hill section: stratigraphic heights vs (*A*) Al2O3, (*B*) total iron (FeO*), (*C*) carbon isotopes of organic matter (δ13Corg), and (*D*) total organic carbon (TOC).Umbaumba section: stratigraphic height vs (*E*) Al2O3, (*F*) FeO*, (*G*) δ13Corg, and (*H*) TOC. δ13Corg 2sd errors are smaller than the thickness of the points. TOC 2sd errors are ≤ 0.06 %.

In the Bruce’s Hill section, above S2, the sedimentary rocks show an immediate and concurrent increase in siliciclastic and Fe-rich materials. Thick sandstones abruptly appear with S2 and decline in thickness and abundance up-section over the next 8 m (Fig. 2). Siliciclastic beds above S2 show evidence for erosive bases, cross-bedding, and abundant lensing, speaking to persistent current activity (Figs. 1*E* and 2 and *SI Appendix*, Fig. S7). Alternating pure chert beds show plastic behavior when eroded into and downward injection of sand, indicating that they were still soft during sandstone deposition (*SI Appendix*, Fig. S7*B*). This may be explained by relatively high sedimentation rates that did not permit enough time for the white cherts to lithify before the next sandstone was deposited. Fe-bearing minerals are predominantly siderite, with some hematite, magnetite, pyrite, stilpnomelane, and, potentially, greenalite (*SI Appendix*, Figs. S11–S13). Siderite itself occurs as rounded <3 µm grains, or larger euhedra (>20 to 30 µm); again, siderite is always associated with organic matter. The siderites do not show evidence for sedimentary reworking, and siderite-rich beds exhibit transitional contacts rather than clear bedding. Hence, we suggest a diagenetic origin. Sub-micron-sized hematite is most common above the second fallback layer at 460 cm where Fe concentrations are the highest (Fig. 4*B*). Here, hematite and larger magnetite occur within a granular jasper bed (*SI Appendix*, Fig. S11). Minor hematite also occurs within siderite-rich beds. Submicron, randomly orientated laths of possible greenalite occur in at least one thin section in association with rounded siderites (*SI Appendix*, Fig. S13).

### Geochemistry.

**Fig. 5.**
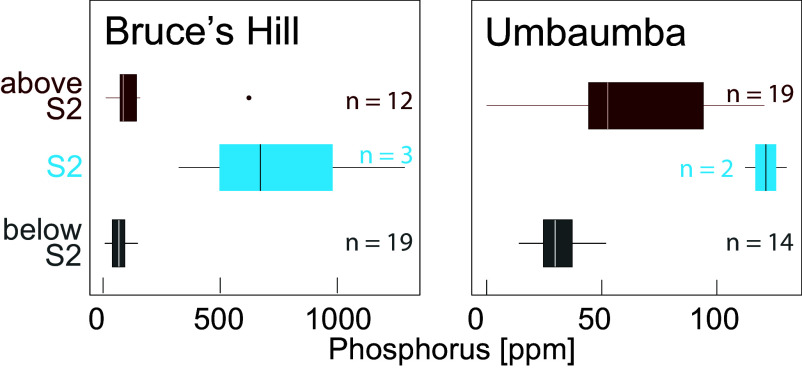
Phosphorus abundance of cherts from below, within, and above S2.

Al_2_O_3_ correlates with Cr, Zr, and Ti and tracks the input of clay-rich terrigenous material (31) (*SI Appendix*, Fig. S14). Below the impact horizon, Al_2_O_3_ abundances are higher for the Bruce’s Hill (2.7 ± 1.5 wt.%) than for the Umbaumba section (0.7 ± 0.6 wt.%) (Fig. 4 *A* and *E*). These values mirror abundances in the shallow-water and shelfal facies, respectively, of the Buck Reef Chert in the Hooggenoeg Formation (37). In the Umbaumba section, there is a marked uptick in Al_2_O_3_ above S2 (Fig. 4*E*, 2.2 ± 1.0 wt.%, *P*-value of 22 × 10^−5^), reflecting a temporary increase in siliciclastic sediment influx before a return to previous values in ferruginous sediments. In the Bruce’s Hill section, there is no statistically significant change across S2 (2.1 ± 1.0 wt.%, *P*-value of 0.75, Fig. 4*A*), although postimpact rocks show a bimodal distribution of Al_2_O_3_ abundances that reflect the alternation of siderite/hematite-rich and siliciclastic-rich beds (Fig. 1*E*). The siliciclastic-rich layers have higher Al_2_O_3_ (3.4 wt.%) than most pre-S2 samples. Importantly, P concentrations correlate with siliciclastic sediment input, especially in the Umbaumba section, and with spherules abundance. P concentrations prior to the impact are low in the Umbaumba (30 ± 10 ppm) and the Bruce’s Hill sections (68 ± 40 ppm, Fig. 5) compared to modern sedimentary settings (e.g., mudstone = 1,135 ppm) (38). P abundances are the highest within S2 (Bruces Hill: 671 ± 488 ppm, Umbaumba: 121 ± 12) but remain slightly elevated above S2 (Bruces Hill: 91 ± 41 ppm, Umbaumba: 46 ± 25). The highest concentration of P (1,300 ppm) was measured in the third sandstone bed within the Bruce’s Hill section, which shows the highest spherule abundance (~10%).

FeO* can track changes in Fe content of the water column, provenance, and volcanic or hydrothermal activity (31, 37). For both sections, FeO* values below S2 are relatively low (Bruce’s Hill: 0.7 ± 0.4 wt.%, Umbaumba: 0.4 ± 0.4 wt.%, Fig. 4 *B* and *F*). In the Bruce’s Hill section, mean FeO* values increase sixfold to 4.0 ± 3.0 wt.% (max 13.1 wt.%) above the impact layer (*P*-value of 2.4 × 10^−9^, Fig. 4*B*). In the Umbaumba section, there is no immediate change in FeO* (1.0 ± 2.0 wt.%, *P*-value of 0.8) until the siliciclastic sediment influx recedes (Fig. 4*F*). Then, FeO* values increase up to 17-fold (7.4 wt.%). Both sections show slight positive Eu anomalies [Eu/Eu* = Eu_SN_/√(Sm_SN_ × Gd_SN_); SN = shale-normalized], indicating some hydrothermal influence (*SI Appendix*, Figs. S15 and S16). Values before the impact are similar to those after the impact (Bruce’s Hill: 1.53 ± 0.16 to 1.68 ± 0.38 wt.%, Umbaumba: 1.49 ± 1.45 to 1.45 ± 0.15 wt.%; *P* values > 0.05 for both). Proxies for relative mafic/felsic contribution (Cr/Zr) do not show any statistically significant changes for the Umbaumba (11.4 ± 7.5 to 9.7 ± 9.9; *P* = 0.18) or a slight shift to a more felsic provenance in the Bruce’s Hill section (7.4 ± 3.1 to 6.4 ± 3.4; *P* = 0.006; *SI Appendix*, Fig. S15) across S2. These values indicate a mixed felsic and mafic provenance. Finally, the vast majority of samples show no negative Ce anomaly, although a weak negative Ce anomaly is seen in one sample from each section (*SI Appendix*, Figs. S15 and S16).

### Carbonaceous Matter: Carbon Isotopes and TOC.

δ^13^C_org_ ranges from −27.5 to −36.4‰ and varies stratigraphically at the cm-scale. Metamorphism, migration of hydrocarbons, and equilibration with carbonates had only a minor effect on the carbon isotopic signatures (*SI Appendix*). These values and variability are similar to previous analyses for carbonaceous cherts of the Onverwacht and Fig Tree groups (29, 37, 39–41) and are consistent with isotopic fractionation during carbon fixation via the Calvin and/or Wood–Ljungdahl pathways.

**Fig. 6.**
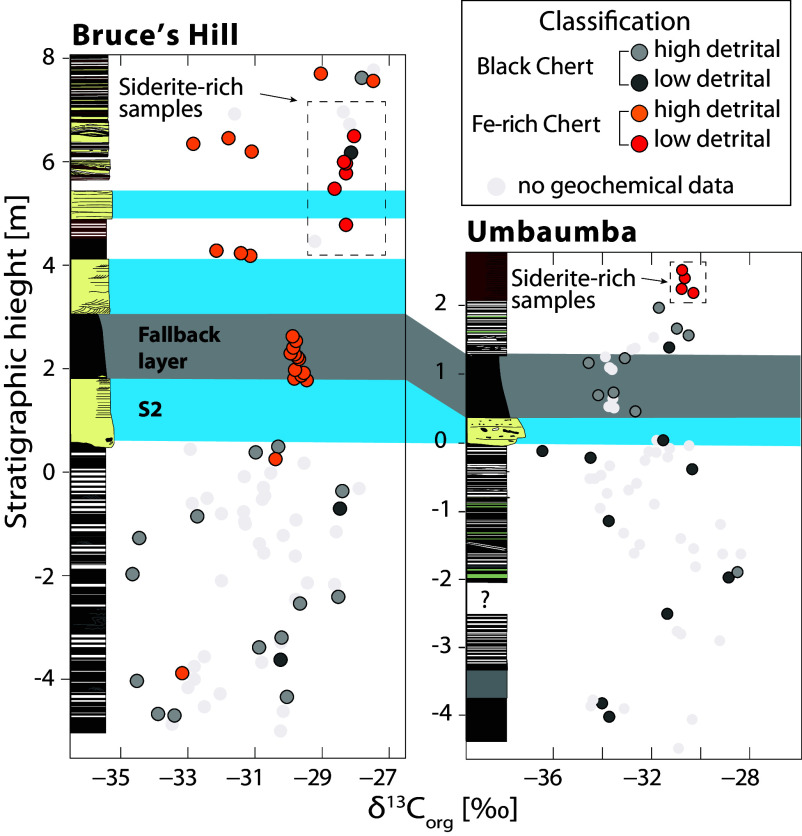
Carbon isotopes of organic matter (δ^13^C_org_) data classified based on their geochemical composition. High-detrital samples have Al_2_O_3_ > 1.5 wt.%; Fe-rich cherts have total iron (FeO*) > 1.5 wt.%. See *SI Appendix*, Fig. S14, for more detail.

δ^13^C_org_ values show strong variability below the spherule bed in both sections (Fig. 4 *C* and *G*), with values ranging from −36.4 to −28.2‰ (mean of −31.8 ± 2.0‰) in the Umbaumba and −34.7 to −27.5‰ (mean of −30.8 ± 1.7‰) in the Bruce’s Hill section. In contrast, the fallback layers show a much narrower distribution (Umbaumba: mean of −33.5 ± 0.5‰; Bruce’s Hill: mean of −29.8 ± 0.1‰). In the fallback layer of the Bruce’s Hill section, δ^13^C_org_ values display a moderate negative trend to lower δ^13^C_org_ values by ~0.5‰ (r^2^ = 0. 35; *P*-value = 0.025; Fig. 4*C* and *SI Appendix*, Fig. S8). The negative trend is, at best, only weak for the Umbaumba section (r^2^ = 0.11; *P*-value = 0.323; Fig. 4*G* and *SI Appendix*, Fig. S8). Above the fallback layers, δ^13^C_org_ values shift toward heavier and more stable values for the Umbaumba section, especially within the ferruginous cherts at the top of the section (-30.6 ± 0.3‰) (Figs. 4*C* and 6). In the Bruce’s Hill section, δ^13^C_org_ values become bimodal (Figs. 4*G* and 6): high FeO*/low Al_2_O_3_ samples show consistently heavier and more stable values (−28.3 ± 0.2‰) compared to low FeO*/high Al_2_O_3_ samples (−31.6 ± 0.6‰). The high Al_2_O_3_ values for the latter reflect a higher influx of debris, likely including older organic matter reworked by currents.

TOC values range from 0.04 to 0.77 wt.% (Fig. 4 *D* and *H*), similar to previous analyses for carbonaceous cherts of the Onverwacht and Fig Tree groups (29, 37, 39–41). In the Umbaumba section, TOC values range largely between 0.1 and 0.2 wt.%, with the exception of higher TOC values in a black chert bed at the base (0.36 ± 0.05 wt.%), the fallback layer (0.26 ± 0.03 wt%), and siderite-rich cherts at the top of the section (0.28 ± 0.05 wt.%). In the Bruce’s Hill section, samples show more scatter in their TOC values, although higher average TOC values can still be seen at similar stratigraphic locations at the base of the section (0.26 ± 0.11 wt.%) and the fallback layer (0.27 ± 0.03 wt.%). Above S2, TOC values are bimodal with samples with higher detrital influx showing higher TOC (0.28 ± 0.05 wt.%) than those with low detrital influx (0.16 ± 0.07 wt.%). A modest covariation (r^2^ = 0.24 and 0.23 for Bruce’s Hill and Umbaumba, respectively; *SI Appendix*, Fig. S17) exists between C-isotopic composition and TOC covariation. TOC, in turn, reflects sediment composition, with high detrital samples tending to have higher TOC and lower detrital δ^13^C_org_ values.

### Carbonate Carbon Isotopes.

**Fig. 7.**
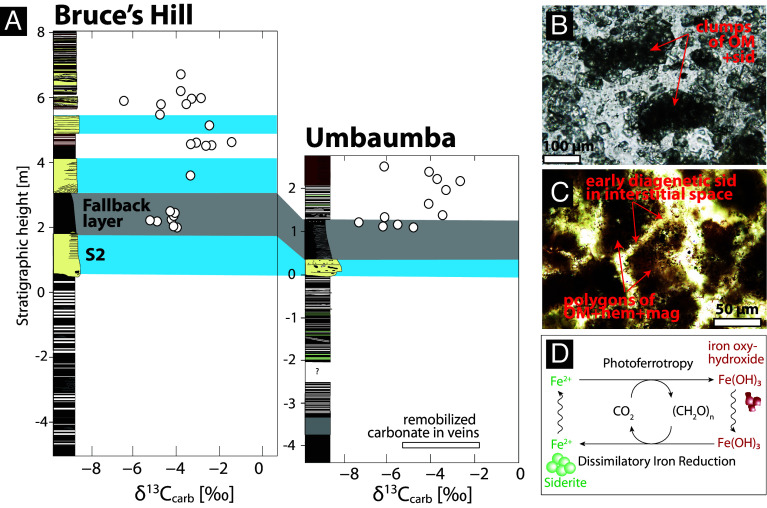
(*A*) Carbon isotope analyses of carbonate (δ^13^C_carb_). (*B*) Thin section image of ferruginous chert above S2 in the Umbaumba section showing clumps of siderites and organic matter. (*C*) Thin section image of Fe-rich sample above the second fallback layer in the Bruce’s Hill Section. Sid = siderite, hem = hematite, mag = magnetite, OM = organic matter. Also see *SI Appendix*, Figs. S11–S3. (*D*) Conceptual model of microbial iron cycling and associated mineral precipitation (46).

δ^13^C_carb_ range between −7.3 and −1.8‰, also variable at the cm-scale (Fig. 7*A*). Some remobilization of carbonate in veins is observed below the S2 spherule bed in the Umbaumba section, as well as within small veins that cross-cut siderite-rich horizons above S2. There is no correlation between δ^13^C_carb_ and δ^18^O_carb_ (*SI Appendix*, Fig. S17). The values suggest a carbon source variably depleted in ^13^C relative to contemporaneous seawater. The background δ^13^C_DIC_ range is actually not well constrained locally as there are no penecontemporaneous shallow water limestone or dolomite beds in the studied sections. However, carbonate veins into underlying komatiites of the Mendon Formation have δ^13^C_carb_ values of ~2‰ (42) and dolomites ~250 m above S2, and 3.24 Ga rocks of the middle Fig Tree Group (34), have values of 1.5 ± 1.5‰ (43) similar to broadly contemporaneous carbonate beds observed globally (44, 45).

## Discussion

### Environmental Effects of the S2 Impact.

#### Environment prior to the impact.

Both sections were deposited in relatively shallow water. Low Fe abundances suggest deposition above the Fe-chemocline in both sections. The Bruce’s Hill section was affected by weak to moderate current and/or wave activity, in an environment with access to siliciclastic debris. In contrast, the Umbaumba section largely lacks current structures and siliciclastic detritus. Rare chert-plate conglomerates and intraclasts in sandstones have previously been interpreted as storm deposits (31). Overall, both sections were deposited above storm wave base, with Bruce’s Hill perhaps in shallower water than the Umbaumba section.

#### Tsunami.

In both sections, impact-derived spherules are located in beds that are characterized by short-lived, atypically high-energy events in sequences that are otherwise dominated by weak (Umbaumba) or moderate energy currents (Bruce’s Hill). These high-energy events behaved like surges: They initially eroded into the sea floor, deposited ripped-up material, and then declined in current energy (47). The normally graded fallback layers appear to be a continuation of this trend, reflecting the settling of suspended organic matter and fine-grained siliciclastic materials ripped up from the sea floor, as well as dust particles and meteoritic material released by the impact. Clearly, there is an intimate association between these high-energy deposits and the impact-derived spherule deposition. Hence, similar to other Archean spherule beds (17–20, 47), we interpret these deposits to reflect impact-initiated tsunami deposits.

The features of the S2 spherule beds are similar to those of modern tsunami deposits. In addition to eroding and depositing sediment as tsunamis inundate coastlines, tsunamis generate powerful backwash flows. Sedimentological characteristics of tsunami deposits indicate a highly variable interplay of processes, including oscillatory flows, lower and upper flow regime currents, combined flows, sediment suspension, and sand injection (48). As a consequence, tsunami deposits show a diversity of features but are commonly identified by their basal erosive surface, anomalously coarse composition in comparison to surrounding sediments, abundance of intraclasts, and fining-upward trend, such as seen after the 2004 Indian Ocean Tsunami (48, 49) and similar to those of the S2 spherule bed. The presence of three spherule-bearing, thick sandstones in the shallower water section (Bruce’s Hill) offers the possibility that multiple high-energy currents passed through the location. This is not unusual for tsunami deposits and may indicate multiple backwash deposits generated by a tsunami wave train. Alternatively, the observed pattern could result from the reflection of the tsunami wave by a land mass or traverse of the tsunami wave across the globe.

These deposits reveal a tight timeline of events. Spherule deposition must have occurred immediately prior to or concurrent with the deposition of the tsunami deposit. After the impact, the rock vapor cloud within which the spherules formed expanded at speeds between 9 to 19 km/s (50). Hence, even at distal locations the spherules settled within hours of the meteorite impact and arrived at the site of deposition before the tsunami, which traveled at a significantly slower speed (~800 km/h in deep-water). By the time the fallback layers were deposited, spherule deposition had already ceased. The deposition of the fallback was also rapid. In the Umbaumba section, the largest grain (56 µm) at the top if the fallback layer would have taken as little as 20 h to settle through a 200 m water column based on Stoke’s Law, assuming no resuspension. Altogether, the spherule beds and fallback deposits (1.3 to 5 m of strata) were likely deposited within no more than a few days—a geological instant. In this limited time period, the impact-initiated tsunami ripped up the sea floor, disturbed coastal benthic biosystems, mixed the water column, washed debris from coastal areas into the sea, and caused turbid conditions.

#### Ocean mixing.

Both spherule beds show an increase in FeO* above the spherule bed. An increase in Fe has also been observed above other Archean impact layers (51, 52) and may relate to impact-initiated mafic volcanic activity (51), uplift and erosion of mafic volcanic rocks (51), increase in hydrothermal activity (51), impact-induced tectonic changes (19) that caused deepening, or mixing of the ocean by the tsunami (52).

Several studies suggest that impacts may have (re)activated volcanism (51, 53, 54) or caused the uplift of mafic crust (51). However, the lack in change of Cr/Zr across S2 in both sections suggests that there was no impact-induced relative increase in mafic volcanic activity and/or uplift and erosion of mafic crust. In addition, there is an increase in Fe even if controlled for siliciclastic influx, i.e., FeO*/Al_2_O_3_ values increase above S2, suggesting that the increase in Fe is not purely related to an increase in sedimentation. Impacts have been shown to generate hydrothermal fields at the impact site (53), which may emit Fe^2+^. Before the impact, the cherts have weak positive Eu anomalies (*SI Appendix*, Figs. S15 and S16) (31), consistent with Archean seawater having a higher hydrothermal input, driven by the higher heat flux from the mantle (54). The lack of change in Eu/Eu* values across S2 suggests that any impact-induced hydrothermal systems were only local and not global. Deepening may have caused the sections to pass through the chemocline into Fe-enriched deeper waters, possibly due to impact-induced tectonic changes with the onset of Fig Tree deposition (19). However, neither section shows any sedimentological evidence for immediate deepening after the impact, while long-term deepening is likely (55). In the Bruce’s Hill section, cross-laminations and erosive surfaces are abundant above S2 (Figs. 1*E* and 2 and *SI Appendix*, Figs. S3 and S7), and no deep-water Bouma sequences could be identified. In the Umbaumba section, evaluation is difficult since only 80 cm of ferruginous chert is preserved, above which there is no more outcrop. This 80 cm section shows a similar grain size to much of the section below S2 (*SI Appendix*, Fig. S15), and cross-laminations indicate some current activity. Importantly, FeO/Al_2_O_3_ ratios of overlying deep-water mudstones of the lowermost Fig Tree Group (33, 56) are lower than those seen above S2 in our sections.

Based on these data, the most parsimonious explanation for the increase in FeO* is the mixing of Fe^2+^-enriched deeper waters with surface waters by the passage of an impact-induced tsunami. As detailed above, there was a Fe-rich deep ocean during Mendon time (31). The tsunami likely reached below the chemocline, bringing Fe-rich deep waters into the otherwise Fe-depleted upper water column.

#### Ocean evaporation.

The pseudomorphs within and above the fallback layer likely formed from brines. Type 1 pseudomorphs within the top of the layer and above formed authigenically within the sediment itself as they show no evidence of abrasion, no preferred orientation, and were not in hydrodynamic equilibrium with the surrounding sediment. Hence, an origin from reworking of rock-forming hexagonal minerals is unlikely. While Type 2 pseudomorphs have a similar appearance, they show a preferred orientation parallel to bedding and no or only minor evidence for reworking. While we cannot exclude the possibility that Type 2 crystallites formed by reworking of Type 1 crystallites from the top of the fallback layer, it is also possible that they formed in the water column itself and settled onto the sea floor. Such evaporative rafts are common in modern brine pools (57).

The original composition of these pseudomorphs can be further constrained. Pseudohexagonal sedimentary/early diagenetic minerals such as barite, tourmaline, apatite, and gypsum appear unlikely: Barite and tourmaline are common within the central greenstone belt as primary minerals (58, 59) and are hard to dissolve; primary apatite is present within the fallback layer; gypsum, while easy to dissolve, should still show its characteristic swallow-tail twinning, which is conspicuously absent. Pseudohexagonal crystals of similar habit and similarly replaced by macro- and microquartz have been identified as nahcolite in the evaporitic facies of the Buck Reef Chert (60). Aragonite was also recognized as a primary evaporite in late Archean and Paleoproterozoic sections (61, 62). As a result of high pCO_2_ of the Archean atmosphere and high HCO_3_^−^ levels in Archean oceans, it has been proposed that evaporative sequences would have precipitated aragonite first, followed by nahcolite (60–62). We hence propose that these pseudomorphs most likely represent aragonite or nahcolite.

These types of crystallite pseudomorphs are reported for the first time from the Mendon Formation (31), and those observed in this study are exclusively located within and immediately above the impact-related fallback layer in the Umbaumba section. If the crystals indeed represent evaporitic minerals, then their first occurrence immediately above the S2 layer within previously subaqueous sections suggests a substantial change in environment. Previous modeling has shown that large impacts can cause heating of the atmosphere, through rock vapor and melt ejected from the crater site, as well as the greenhouse effect of gases emitted into the atmosphere (2). As a consequence, boiling of the upper water column and evaporation of meters to tens of meters of sea water are possible for an impact the size of S2 and would have lasted for a little more than a year (2). It is thus conceivable that the two types of pseudomorphs grew from brines as a result of impact-induced partial ocean evaporation. They formed as interstitial evaporites and, potentially, as pelagic crystals that grew as rafts in the uppermost part of the water column where evaporation was highest and settled to the sea floor.

The presence of Type 1 interstitial crystallites in the unusually debris-rich meter above the fallback layer in the Umbaumba section may suggest that evaporitic conditions persisted during this time and that those sediments were deposited before the evaporated water rained back out. The higher siliciclastic input and current energies in that part of the section may thus relate to an increase in weathering, erosion, and sediment influx due to the postimpact hothouse and/or the relative shoaling due to the evaporation of the uppermost water column. While it is notable that these crystallites were not observed in the Bruce’s Hill section, this location was generally characterized by higher-energy currents and the several thick spherule-bearing sandstones show erosive bases. Any evidence for ocean evaporation may have been eroded.

#### Influx of phosphorous.

Both sections show an increase in P with the impact, associated with the increase in siliciclastic detritus and the deposition of spherules. Debris would have been flushed into coastal areas as a result of tsunami backwash and terrestrial environments would have been subject to aggressive weathering in the impact-induced hothouse. This would have increased nutrient availability, assuming that at least some of the P-bearing minerals such as apatite may have hydrolyzed to release bioavailable P. The high P abundances in spherule beds also suggest a meteoritic component to the increase in P. Carbonaceous chondrites, the proposed bolide type for S2 (25), can contain up to 0.107 wt.% P (63), hence as much as 363 Gt P for S2. Much of this P would have been in a reduced state and thus bioavailable (64, 65).

### Biological Effects of the S2 Impact.

#### Biosphere prior to the impact.

The nature and diversity of early Archean life remain uncertain. For most of Earth history, the δ^13^C_org_ in marine sedimentary rocks has been interpreted in terms of CO_2_ fixation by photosynthetic organisms using the Calvin-Benson cycle (29, 37, 66–70). In Paleoarchean rocks, it is possible that chemoautotrophs using the Wood–Ljungdahl pathway were at least locally dominant, given the antiquity and observed C-isotopic fractionation associated with this metabolism (71), and the presence of environments conducive to such organisms, e.g., hyperthermophiles at vents (72). Photoferrotrophs likely existed in the Paleoarchean (73), but were unlikely dominant in the shallow water column of the Mendon Formation due to the dearth of Fe^2+^. Whether or not cyanobacteria existed at the time of the impact is unclear. Satkoski et al. (36) have controversially argued that O_2_ produced by oxygenic photosynthesis accounts for the scarcity of iron in shallow water facies of the Fig Tree basin. While some molecular clocks suggest an early Archean origin of Photosystem II, required for oxygenic photosynthesis (66), molecular clocks place the origin of crown group cyanobacteria long after the S2 impact event (67). It is possible that some form of primordial oxygenic photosynthesis was present during the Paleoarchean (66), but it might well have been restricted to fresh waters where electron donors other than water were limited; oxygenic photoautotrophs may not have competed well against anoxygenic competitors in marine waters where alterative electron donors were abundant (68–70). In addition, available geochemical evidence constrains the oxygen content of Paleoarchean marine waters to exceedingly low values (74), militating against free O_2_ and iron-based chemoautotrophy. Whatever the nature of primary producers, however, rates of primary production were low, limited by phosphorus availability (69, 72, 75, 76), electron donors (77), or both. At the same time, remineralization of organic matter in the water column or sediments would have been inefficient and perhaps largely restricted to fermentation followed by methanogenesis, given the limited availability of oxidants for respiration (75).

The variable and light δ^13^C_org_ values before the S2 impact in both sections suggest that either a metabolism was active that introduced isotopic heterogeneity or that diverse metabolisms in the local ecosystem imparted variable degrees of fractionation. The lightest δ^13^C_org_ are at or below the limit for the Calvin-Benson Cycle and may hence indicate operation of the Wood–Ljungdahl pathway (78–80). Similar patterns of δ^13^C_org_ variation in other Archean and Proterozoic rocks (37, 39, 81–83) have been interpreted in terms of photosynthetic fractionation via the Calvin-Benson cycle, modified within the sediments by secondary processes such as methanogenesis, methanotrophy, and acetogenesis via the Wood–Ljungdahl pathway. While such an interpretation makes sense, the low TOC content of isotopically heavy carbonaceous matter makes them prone to contamination by, for example, detrital organic carbon from the erosion of older black cherts.

#### Biological consequences of the impact.

**Fig. 8.**
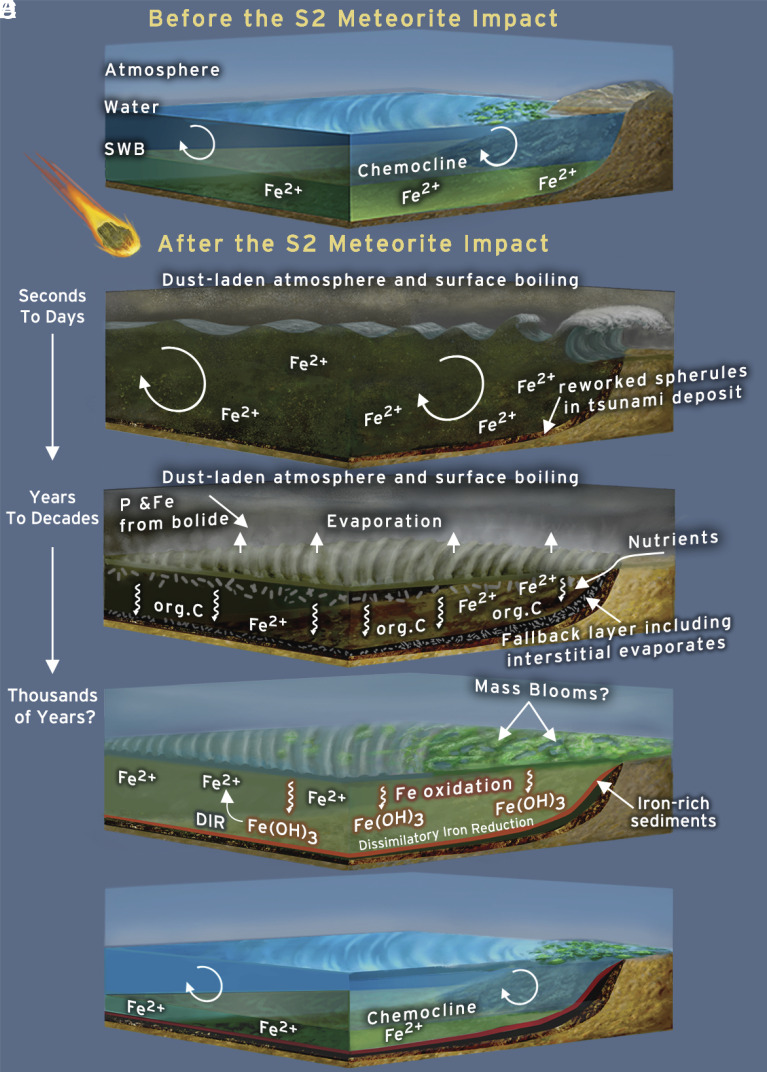
Schematic representation of possible effects of the impact on environments and life. (*A*) Environment prior to the impact. Water column is defined by a Fe^2+^-enriched deep and Fe^2+^-depleted shallow water column. Life (represented by green patches) was active, but likely less productive than today. (*B*) The impact initiated a large tsunami that swept across the globe. In the process, it ripped up the sea floor, mixed the water column, and caused turbid waters. The emission of dust and particles into the atmosphere by the impact caused global darkness, which may have lasted years to decades. (*C*) Impact heating reaches its peak, causing partial ocean evaporation oceans, the precipitation of evaporite minerals within the water column and the sediment, and an increase in weathering and erosion of exposed landmass. This would have lasted for up to a few years (2). (*D*) Once the dust had settled and the water rained back into the oceans, conditions may have been beneficial to life. P and Fe concentrations increased due to contributions from the bolide itself, an increase in weathering and erosion, and mixing of Fe-rich deep waters into shallow water. Especially Fe-based metabolisms may have benefited from the increase in Fe in the shallow water column. (*E*) The environment returns to preimpact background values until the next impact. DIR = dissimilatory iron reduction, org. C = organic carbon; Fe(OH)_3_ = iron oxyhydroxides.

It might be assumed that all physical consequences of the S2 impact must have been disastrous. Life was exposed to multiple environmental challenges, including the ripping up of sea floor in shallow-water environments by the tsunami, evaporation of the uppermost layer of the ocean due to heating, and darkening from silicate dust injected into the atmosphere and suspended particles in the water column. All of the above may well have had a negative effect on microbes after the S2 impact, but postimpact strata strongly suggest that life not only persisted but rebounded rapidly (Fig. 8).

In the Phanerozoic record, mass die-offs after meteorite impacts are captured in carbon isotopic signatures. For example, the famous K-Pg impact is associated with transient global decreases in δ^13^C_org_ and δ^13^C_carb_ of up to a few ‰ (3, 13). This was likely the result of a drop in marine primary productivity and export, which caused the surface-to-deep carbon isotope gradient to collapse (3, 13). In the Archean, however, the biosphere was likely not productive enough compared to the large size of the dissolved inorganic carbon (DIC) reservoir to generate a strong δ^13^C_DIC_ gradient between shallow and deep-water (72, 84, 85). While both Mendon sections show a weak negative δ^13^C_org_ trend within the fallback layer, this layer formed by the settling of material ripped up from the ocean floor after the passage of the tsunami. The tight clustering of δ^13^C_org_ values in both sections may thus reflect the homogenization of suspended materials by tsunami-induced mixing and rapid burial that limited secondary metabolisms. The negative δ^13^C_org_ trend correlates with a decrease in grain size (*SI Appendix*, Fig. S8) and thus may also reflect size sorting of particles with different δ^13^C_org_ values. Size-dependent δ^13^C_org_ values of organic matter are observed in modern settings and may reflect different origins or different degrees of degradation (86). In addition, the suspended particles may have mixed with concurrent biomass. The mean δ^13^C_org_ values differ between sections, likely reflecting distinct local detrital C contributions. Different local source terranes are supported by different detrital zircon signatures of the S2 beds in the two sections (87). While both sections show higher TOC within the fallback layer (Fig. 4 *D* and *H*), such high TOC levels are not unusual within the sections and may be due to resedimentation of previously deposited organic matter together with a high sedimentation rate. While we believe that the S2 impact most certainly had a negative effect on phototrophs living in the upper water column, the effect appears to have been short-lived. This appears to stand in contrast to the protracted biological recovery following the Chicxulub impact, but the latter observation refers mostly to animals and exclusively to eukaryotes. Given their huge population sizes, potentially rapid doubling times, and, commonly, metabolic versatility, we would expect recovery among bacteria and archaea to be much faster. Such a short-lived die-off and rapid recovery may not be traceable in the geological record.

The recovery of life would have been fueled by an increase in ferrous iron in the photic zone and enhanced nutrient (especially phosphorous) availability, both indicated by geochemical data. In theory, the influx of Fe may have had two important effects: Fe^2+^ supplied an important electron donor for photosynthesis (photoferrotrophy), and the Fe^3+^ generated in consequence increased the inventory of terminal electron acceptors for respiration (dissimilatory iron reduction, DIR) (88). This is important since DIR is energetically more favorable than other metabolisms (89) commonly cited as being present in the Archean, such as sulfate reduction or methanogenesis.

Our data provide some evidence that microbial iron cycling may have been enhanced due to the influx of Fe from impact-induced ocean mixing. First, there is a shift in δ^13^C_org_ associated with the deposition of iron-rich sediments, specifically for those samples not contaminated by reworked detritus (i.e., those with low Al_2_O_3_, Figs. 4 and 6). The carbon isotope signature becomes more homogenous and comparatively heavy (Fig. 6), suggesting a connection between the carbon cycle and increase in Fe. More consistent and stable δ^13^C_org_ values may reflect a shift in metabolism, especially since access to CO_2_ was unlimited. Second, the presence of iron oxides, siderite, and other Fe-rich phases, which are highly unusual in shallow-water Paleoarchean environments, requires a mechanism of formation. Several processes could underpin the observed iron oxidation. In principle, Fe^2+^ from anoxic deep waters could have been oxidized to precipitate Fe(III) (oxyhydr)oxide as it upwelled into oxygenated surface waters. The presence of a weak negative Ce anomaly in two Fe-rich samples (*SI Appendix*, Fig. S15) may indicate the local presence of O_2_. However, due to biological and environmental reasons cited above, the low number of samples with negative Ce anomalies, and the fact that such anomalies from the BGB have been attributed to alteration (90), we posit that follow-up work is necessary to evaluate a primary vs. secondary origin. For these reasons, and acknowledging uncertainties, we argue that iron-based photoautotrophy provides the most compelling explanation for the observed iron oxides just above the impact horizon. Siderite is spatially associated with organic matter (Fig. 7 *B* and *C*), largely diagenetic in character, and has negative and highly variable δ^13^C_carb_ values. The combined evidence points to a diagenetic origin, where organic matter is remineralized through the reduction of Fe^3+^ during DIR. This process converts ferric oxyhydroxides into soluble Fe^2+^ using electrons derived from organic matter (Fig. 7*D*). This process is a strong alkalinity pump and drives siderite precipitation. The carbon released from organic matter into pore waters can contribute to the DIC stock. Siderite δ^13^C_carb_ values will hence carry an isotopic remanence of the organic matter, causing negative and highly variable δ^13^C_carb_ values on a small stratigraphic scale (91). It bears mention that spherical siderite and greenalite have been obtained experimentally through DIR (99 to 101). Hence, many researchers regard Archean siderite as an early diagenetic sediment formed through DIR (85, 91–93). Additional siderite may have formed during early metamorphism through the reaction of Fe oxyhydroxides with organic matter (94, 95). Last, while an increase in temperature and silicate weathering certainly occurred, early Earth impacts were unlikely to have triggered abiogenic siderite formation in the water column (96).

## Conclusions

The environmental effects of the S2 meteorite impact, and probably other large early Archean impacts, appear to have had mixed effects on early marine life. Some forms of life were positively impacted while others faced increased challenges. The tsunami, ocean evaporation, and darkness most severely affected phototrophs in surface waters but chemoautotrophs in the lower water column and hyperthermophiles would likely have been less influenced. Deleterious environmental effects would have been short-lived, possibly no longer than a few years to decades, and the biosphere would have recovered rapidly. In the medium term, the influx of phosphorus and the injection of Fe^2+^ into shallow waters initiated a transient mass bloom, especially triggering microbial Fe cycling. This adds to the possibility that giant meteorite impacts, typically seen as agents of destruction and extinction, carried transient benefits to life early in Earth’s history. Our work suggests that on a global scale, early life may have benefitted from an influx of nutrients and electron donors, as well as new environments, as a result of major impact events.

## Samples and Methods

The present study is based on the analysis of two stratigraphic sections of S2 located in the central part of the BGB (*SI Appendix*, Fig. S1*C*) (19, 47). Informally named Bruce’s Hill and Umbaumba, these sections are 7.4 km apart and located in two different structural belts separated by the Granville Grove Fault (*SI Appendix*, Fig. S1*C*). The original distance between these two localities is uncertain.

We measured detailed stratigraphic sections at the cm-scale and collected 214 samples from 5 m below S2 and up to 8 m above S2. The tops of the sections were defined by the end of accessible outcrop, which reflected the transition to recessively weathering mudstones. We obtained thin sections, bulk rock geochemistry, TOC, and δ^13^C_org_ and δ^13^C_carb_ on the same samples where possible. In total, we prepared 101 thin sections for petrographic analyses. We analyzed 83 samples for major, trace and rare earth elements at the GeoAnalytical Lab at Washington State University. To test for stratigraphic changes, we used a two-sample Wilcoxon test between geochemical analyses below and above S2. 152 samples were analyzed for their organic carbon isotopic composition (δ^13^C_org_) at Stanford University and 148 samples for TOC analyses at GeoMark Research. We selected 93 samples for carbonate carbon isotope analysis (δ^13^C_carb_) at in the Stable Isotope Laboratory in the Department of Earth Sciences at ETH Zurich, 45 of which yielded enough carbonate for analysis. Raman analyses were conducted in the Fischer lab at Harvard University. Detailed methodology can be found in *SI Appendix* and all data in Datasets S1–S5.

## Supplementary Material

Appendix 01 (PDF)

Dataset S01 (XLSX)

Dataset S02 (XLSX)

Dataset S03 (XLSX)

Dataset S04 (XLSX)

Dataset S05 (XLSX)

## Data Availability

All study data are included in the article and/or supporting information.
