# Supplementary material for: Effect of a giant meteorite impact on Paleoarchean surface environments and life
Source: Proc Natl Acad Sci U S A. 2024 Oct 21;121(44):e2408721121. doi: 10.1073/pnas.2408721121 (PMC11536127; doi:10.1073/pnas.2408721121)
Supplement: Supplementary file 1 — Appendix 01 (PDF) [file pnas.2408721121.sapp.pdf]

PNAS

Supporting Information for
Effect of a giant Paleoarchean meteorite impact on early surface environments and life

Nadja Drabon, Andrew H. Knoll*, Donald R. Lowe, Stefano M. Bernasconi, Alec R. Brenner, David A. Mucciarone

Corresponding author: Andrew Knoll
Email: aknoll@oeb.harvard.edu

This PDF file includes:

Supporting text
Figures S1 to S18
SI References

Other supporting materials for this manuscript include the following:

Datasets S1 to S5

Methodology

For all samples, weathered parts of the rock were removed utilizing a diamond saw cooled with water. Thin section billets were sent to Spectrum Petrographics (https://www.petrography.com) and Wagner Petrographics (https://www.wagnerpetrographic.com). Major and trace elements analyses were conducted XRF and ICP-MS by the GeoAnalytical Lab at Washington State University. TOC analyses were conducted by GeoMark Research LLC (https://www.geomarkresearch.com).

TOC analyses by GeoMark. We ground 2-10 grams of sample using a mortar and pestle until the generated powder passed through a 60-mesh sieve. The samples were then decarbonated by adding 1N HCl acid to the powder for at least two hours. To remove the acid, we then rinsed the samples with water and flushed them through a filtration apparatus. The filter was then removed, transferred to a LECO crucible, and dried in a low-temperature oven at 110°C for at least 4 hours. We used standards with certified carbon content to calibrate the LECO C230. These standards undergo combustion at 1200°C in an oxygen-rich environment, producing CO and CO_2. The CO was converted to CO_2 through a catalyst, and the resulting CO_2 quantified using an IR cell. Unknown samples were combusted, and their carbon content determined by comparing their response to the standards. To ensure consistent and accurate results, calibration standards were analyzed every 10 samples to monitor instrument performance and precision. Additionally, random and selected samples are re-analyzed to verify data integrity. Acceptable variation in TOC measurements was 3% (s.d.) from the established value.

Organic matter carbon isotope analyses. For carbon isotope analyses, the samples were pulverized with a piston crusher and sieved with a 53 μm mesh. The samples were then treated following the methodology of Durand (1) with 43% HF acid to dissolve the chert and 6N HCl to remove micro-carbonates. Both treatments were followed by multiple cleanings with deionized water. Between 0.16 to 8 mg of treated sample powders were sealed in a tin foil capsule for analysis. Samples were analyzed for their carbon isotope composition with a Thermo Finnigan Delta Plus mass spectrometer coupled with Carlo Erba NA1500 Series 2 Elemental Analyzer via a Thermo Finnigan ConFlo II open split interface. We measured samples at no dilution, 10 psi, and 14 psi helium dilution depending on sample size. Standard measurements of USGS40 (published value of -26.39 ± 0.09 ‰) were -26.27 ± 0.15‰, -26.39 ± 0.06 ‰ and -26.39 ± 0.06 ‰ for three analytical sessions. About 10% of unknowns were replicated and show a mean standard deviation of 0.2 ‰. All $\delta^{13}C_{org}$ values are reported relative to V-PDB (Table 2).

Raman analyses. We used microscopically resolved Raman spectroscopy to analyze polished offcuts from thin sections of selected samples. These allowed for phase identification and geothermometry on carbonaceous matter. We used a Horiba XploRA PLUS confocal Raman microscope equipped with a 532 nm excitation laser, filtered to no more than 25% of its total 150 mW output power. All analyses used an 1800 line-per-mm grating and were calibrated to the 521 cm^{-1} characteristic vibrational mode of monocrystalline silicon (2). Spatial resolution (i.e., spot size) is limited by the numerical aperture of the microscope objective to 1.3 μm horizontally for all reported analyses.
For phase identification, we compared spectra to those from a variety of known samples and from the RRUFF project Raman database (3). For geothermometry of carbonaceous matter, we used the spectrum-dependent peak-fitting technique of Kouketsu et al. (4). All spectra from this study satisfied conditions for their fitting "E," which applies to samples matured to ~300-380°C. We implemented the peak fitting via the MatLab function "fit" with a variable-height linear baseline and a combination of Pseudo-Voigt peaks with unconstrained lineshape between Gaussian and Lorentzian profiles. This fit peaks to the D1-D4 and G bands of carbonaceous matter, as well the ~1060-1240 cm^{-1} features of quartz and ~1085 cm^{-1} features of carbonates as appropriate (Fig. S18). We did not encounter spectra with evidence for hematite (bands at ~290, 608, and 660 cm^{-1}), ruling out contamination of the carbonaceous matter ~1345 cm^{-1} D1 band by the hematite 1308-1320 cm^{-1} feature (5). For all geothermometry analyses, we calculated the peak maturation temperature from the full-width-at-half-maximum of the D1 band: T_{MAX}(°C) = 478 – 2.15*FWHM

(cm^{-1}). Fitting error (i.e., analytical error) was ≤2°C for all spectra, much less than the ~30°C systematic error inherent to all carbonaceous matter geothermometry measurements in this metamorphic range (4). We collected spectra from three to five points on each sample analyzed for geothermometry to sample across any variability in carbonaceous matter textures and stratigraphic layering within each sample, which did not exert any observable effects on the calculated maturation temperature. All spectra from Bruce's Hill (n=3) yielded T_{MAX} between 343-358°C, and those from Umbaumba (n=11) yielded similar values within error, between 351-371°C.

Carbonate carbon isotope analyses: We used previously developed methods to measure the isotopic composition of carbonates (6). Briefly, we filled 12 ml Exetainers (Labco, High Wycombe, UK) with 1 to 20 mg of sample powder and flushed them with pure He. We then reacted the samples with 15 drops of 100% phosphoric acid at 70°C for 72 hours in a ThermoFisher GasBench device. The long reaction time guarantees the complete reaction of siderite (7). The produced CO_2 was analyzed with a ThermoFisher Delta V mass spectrometer.
Measurements using replicated standards demonstrated high long-term reproducibility, with an average precision better than 0.1‰. The instrument was calibrated using international standards NBS19 ($\delta^{13}C$ = 1.95‰, $\delta^{18}O$ = -2.2‰) and NBS18 ($\delta^{13}C$ = -5.01‰, $\delta^{18}O$ = -23.01‰). Isotope values are reported relative to the V-PDB (Vienna Pee Dee Belemnite).

Origin, alteration and metamorphism of organic matter
To assess the origin of the $\delta^{13}C_{org}$ values, the effects of prograde metamorphism, migration of organics, and equilibration with carbonates need to be assessed. Raman geothermometry of carbonaceous matter preserved in cherts demonstrates that both sections reached ~350 ± 30 °C (Fig S18). These results are similar to previous estimates for the central BGB (8, 9) indicating that the organic matter has a thermal maturity equivalent to that of the host rock, suggesting the syngenetic origin of the carbonaceous matter with the host rock.
Metamorphism can lead to the preferential loss of isotopically light carbon compounds by thermal cracking. Within the BGB, this effect is thought to have been <3 ‰ (10) and should have been relatively uniform throughout the stratigraphy. In the two sections analyzed here, the stratigraphic dependence of the $\delta^{13}C_{org}$ values and variability suggests that metamorphism did not cause significant large-scale overprinting. Migration of hydrocarbons was only minor since only a single thin section shows a very small amount of hydrocarbon within a fracture fill. At the same time, laminations at the scale of a few microns are abundantly preserved (Figs. 1F, 3F, S4-S6). Lastly, isotopic exchange with carbonates may lower $\delta^{13}C_{org}$ value, although it would have been relatively sluggish at this low metamorphic grade (10) and samples with carbonates show no clear correlation between carbonate wt.% and $\delta^{13}C_{org}$ (Fig. S17).
Carbonaceous materials show a variety of features but occur most commonly as simple and complex carbonaceous grains, carbonaceous laminations, or reworked carbonaceous chert intraclasts. Carbonaceous laminations show several characteristics similar to modern and fossil microbial mats and films (11–18). Similar to arguments in Hickman-Lewis et al. (15) for microbial mats of the Middle Marker, we ascribe a biological origin because [1] The laminations are strongly non-isopachous in character, with irregular morphologies displaying bifurcating and anastomosing structures around lenses of pure silica or detrital particles (Figs. S4 and S5). These crinkly structures are evident on the small and the large scale. Their topography reaches up to 250 µm in height. [2] Individual laminations, especially the thicker ones (Fig. S4) can be traced for many centimeters, although they are internally composed of much shorter, film-like laminations. [3] The cohesive plasticity of ripped-up fragments of mats is further in support of a biological origin (Fig. S4). [4] Similar to other microbially induced sedimentary structures, the laminations draped and bound detrital particles (Fig. S5B-E). The particles are oriented parallel to subparallel to bedding and rarely show grain-to-grain contact. [5] The carbonaceous laminations mantle detrital layers, essentially biostabilizing them (bottom of Fig. S5B). [6] Carbonaceous laminations appear in a shallow water environment plausible for microbial mats to have flourished in. Similarly, previous studies interpreted carbonaceous laminations within the Mendon Formation as microbial mats (11, 19). While the origin of simple and complex carbonaceous particles is less certain, some of these may reflect reworked microbial mats.

In summary, the carbonaceous matter in the two analyzed sections exhibits features typical of biologically derived material that has undergone low-grade metamorphism.

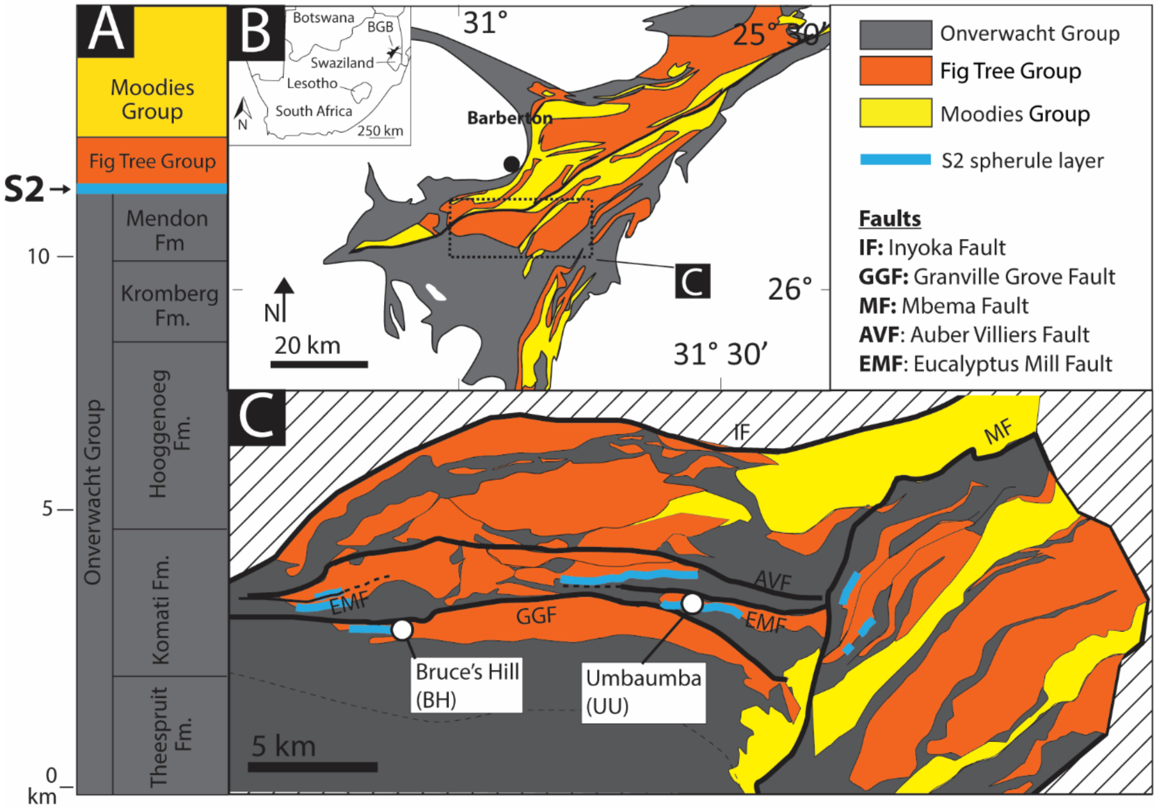

Fig. S1. Stratigraphic column and map of the Barberton Greenstone Belt (20). [A] Stratigraphic column and [B] geological map of the Barberton Greenstone Belt modified from (20). [C] Detailed map of the central BGB modified from Lowe et al. (21) showing the Umbaumba (-25.900883°, 31.005883°) and Bruce's Hill locations (-25.915261°, 30.933052°). Both locations are now within the World Heritage Site of the Barberton Makhonjwa Mountains.

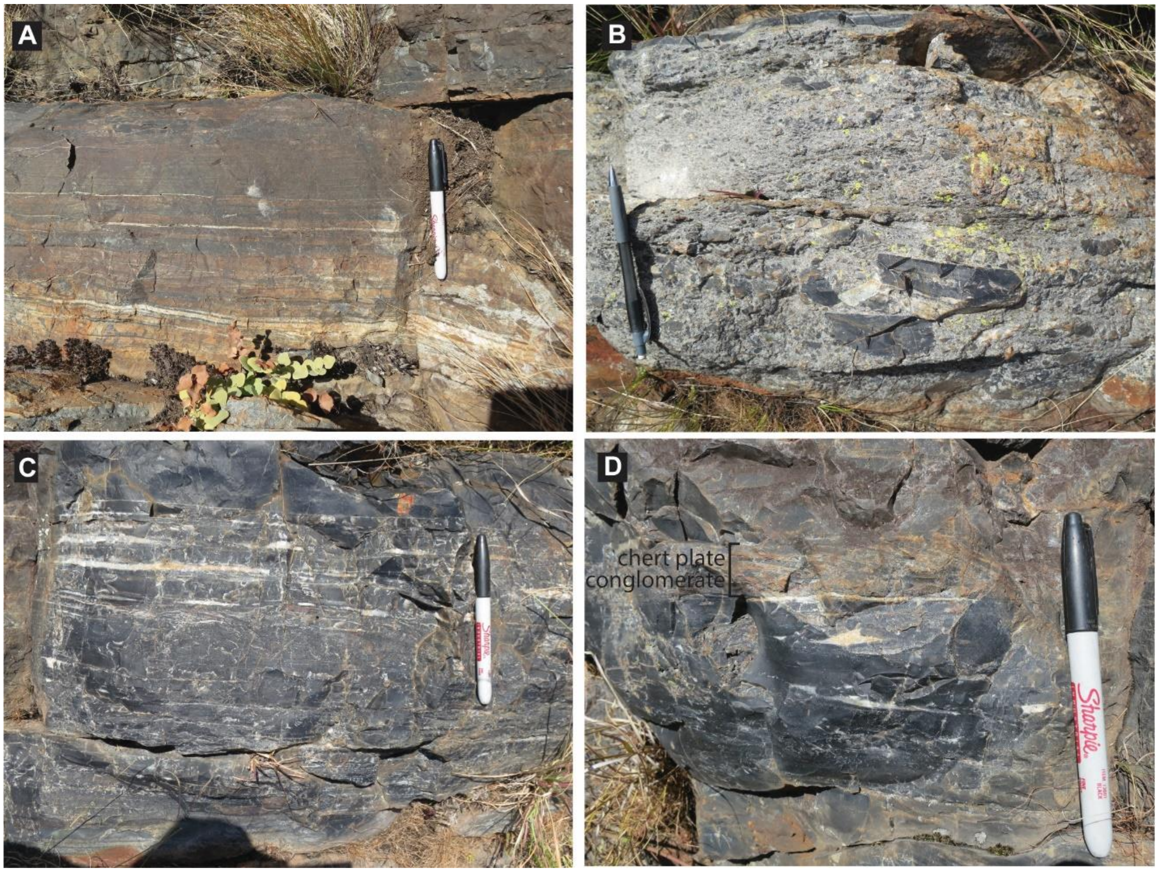

Fig. S2. Outcrop photos of the Umbaumba section. [A] Ferruginous chert above S2. While oxidative weathering gives the rock a red surface appearance, fresh samples look essentially black. [B] S2 spherule bed with large chert clasts in a sandy matrix. [C] Black-and-white banded chert. Due to the freshness of the outcrop many of the translucent, pure chert layers appear black. [D] Black-and-white banded chert and chert plate conglomerate. Please see additional photos in the main part of the manuscript, photomicrographs of ferruginous chert in Fig. S12, and of black-and-white banded and black chert in Figs. S4-S6 and S10.

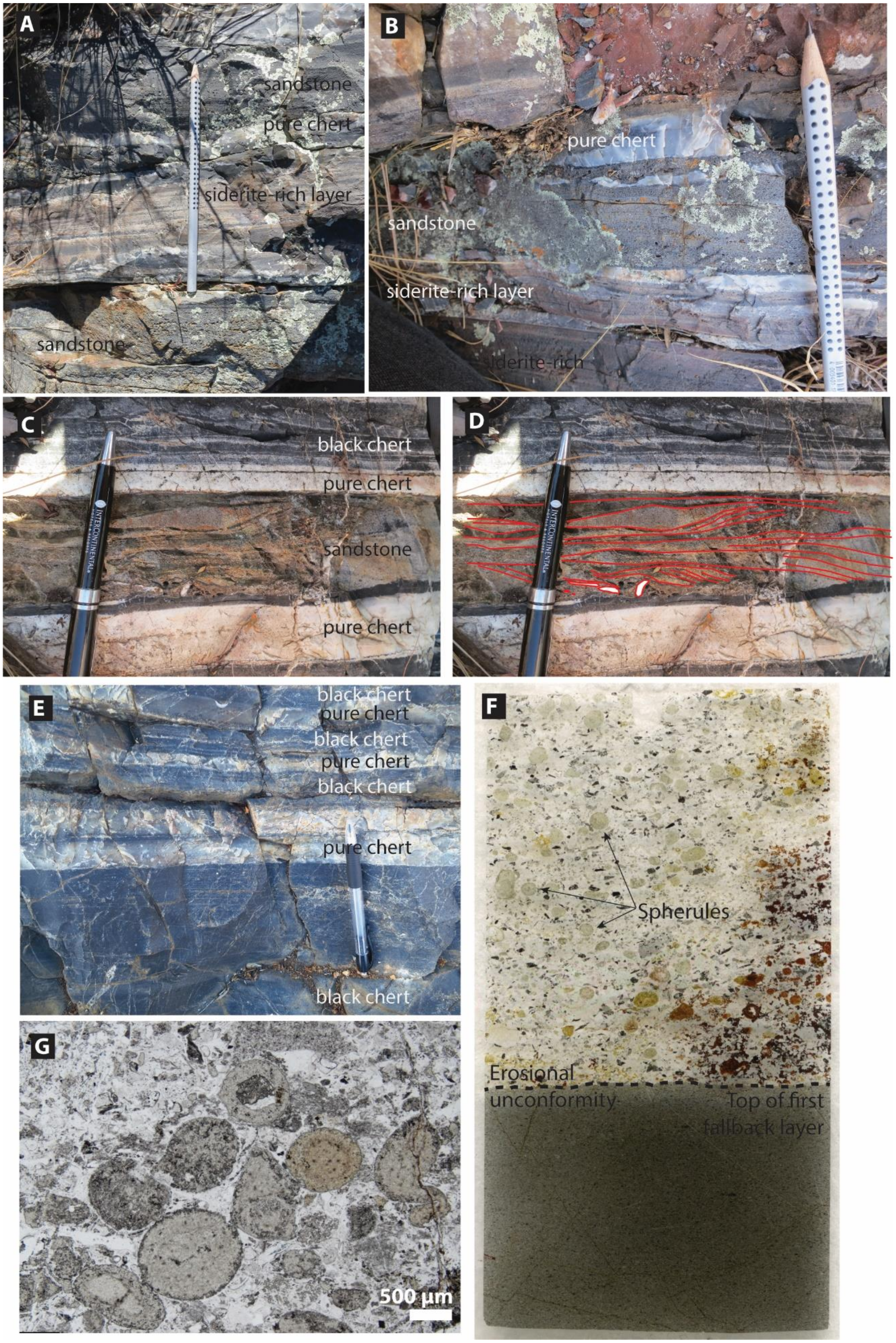
A
sandstone
pure chert
siderite-rich layer
sandstone
B
pure chert
sandstone
siderite-rich layer
C
black chert
pure chert
sandstone
pure chert
D
E
black chert
pure chert
black chert
pure chert
black chert
pure chert
black chert
F
Spherules
Erosional unconformity
Top of first fallback layer
G
500 µm

Fig. S3. Outcrop and thin section photos from the Bruce's Hill section. [A + B] Alternating layers of sandstone, siderite-rich chert, and pure chert above the S2 spherule layer. [C] Alternating cross-laminated sandstone, pure chert, and black chert layers below S2. [D] Same as C but with laminations traced. [E] Black-and-white banded chert below S2. [F] Erosional unconformity into the first fallback layer overlain by the second spherule-bearing sandstone. [G] Spherules of the S2 spherule bed in Bruce's Hill section. See photomicrographs of ferruginous chert in Fig. S12 and of black-and-white banded chert in Figs. S5 and S6.

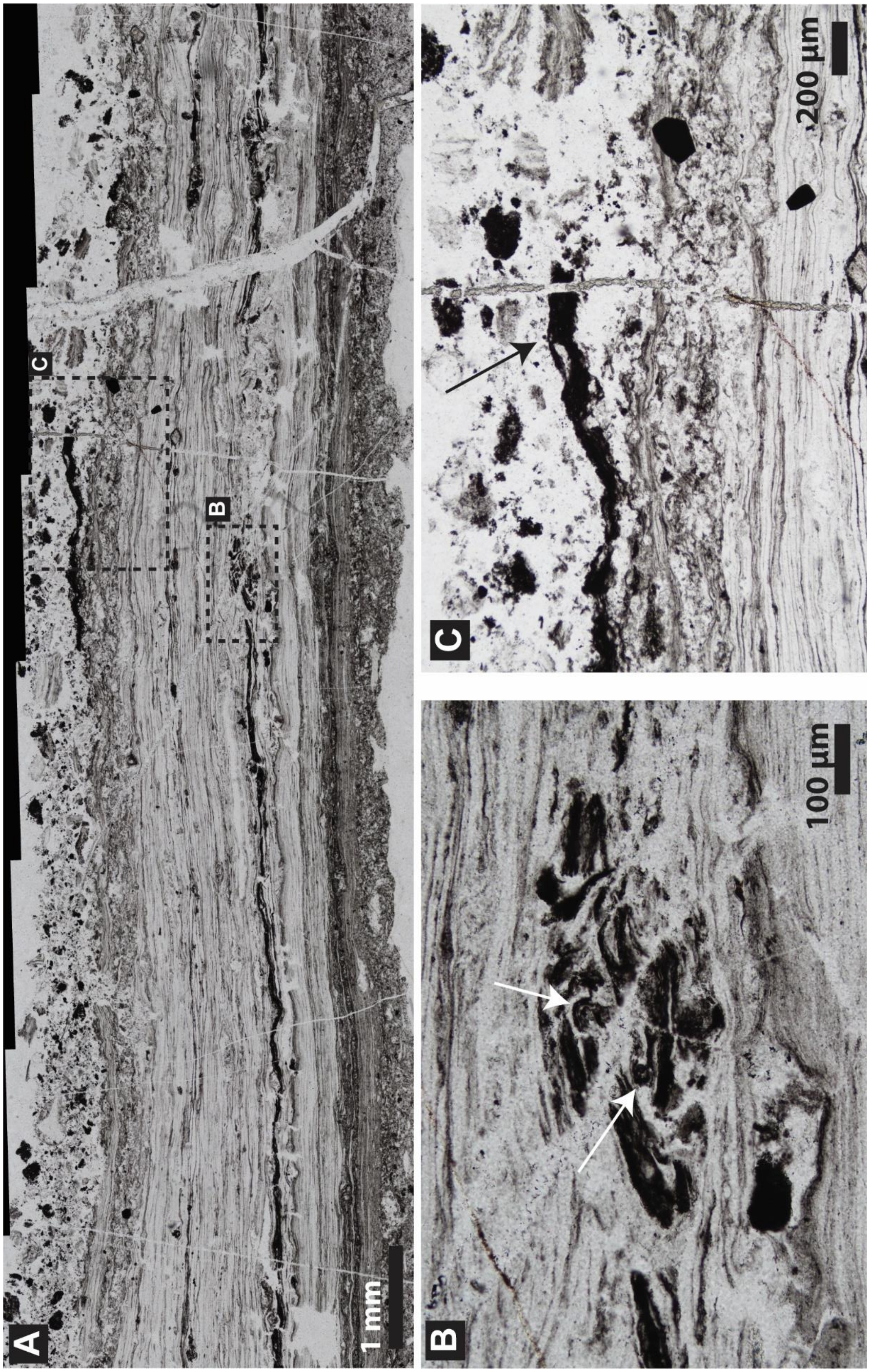
A
B
C
1 mm
100 µm
200 µm

Fig. S4. Carbonaceous laminations in the upper Mendon Formation from the Umbaumba section below the S2 spherule bed (Noffke et al., accepted)**.** [A] Variable carbonaceous laminations overlain by a detrital layer. The laminations are separated by layers of pure chert and often contain outsized carbonaceous particles. Laminations appear to show a bimodal thickness distribution, with thinner laminations between 2-10 µm and thicker laminations 20-100 µm in thickness. Thick laminations can extend across the entire thin section, but are internally made up of shorter, thin laminations. They are relatively smooth with little surficial relief. The thicker laminations show evidence for cohesion: [B] Zoom into small area showing roll-up structures (white arrows) suggestive of cohesion. The thick lamination is clearly composed of stacks of individual, thinner and less continuous laminations. [C] Zoom into thick carbonaceous lamination that is in the process of being eroded, also exhibiting internal integrity. Large, dark clasts in the detrital layer at the top of [A] may reflect reworked fragments of microbial mats. Thin laminations are, albeit their low relief, non-isopachous and show abundant anastomosing and bifurcating characteristics.

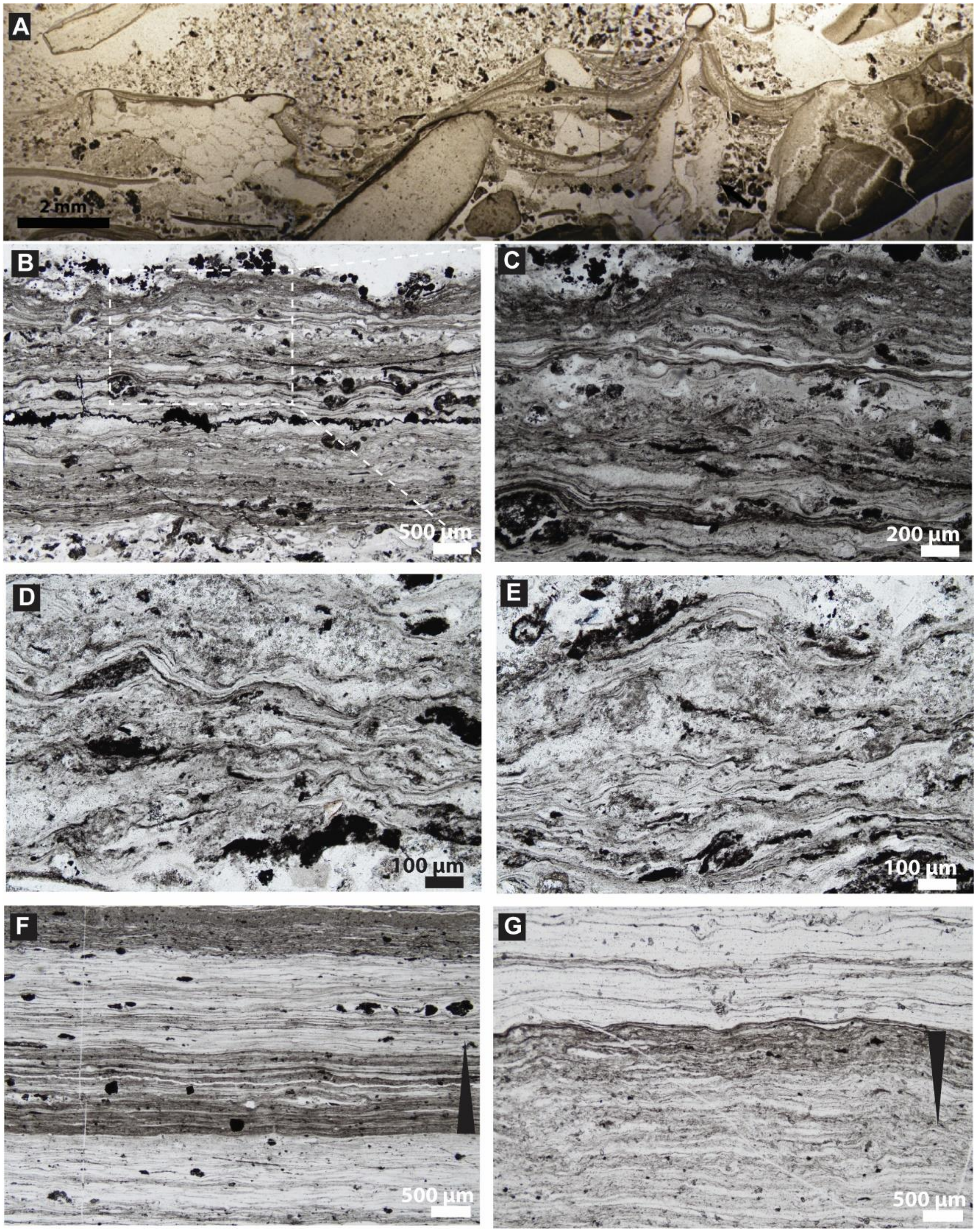
A
2 mm
B
500 µm
C
200 µm
D
100 µm
E
100 µm
F
500 µm
G
500 µm

Fig. S5. Crinkly and flat-laminated morphologies of carbonaceous laminations in the upper Mendon Formation. [A] Clasts draped by carbonaceous laminations, which were interpreted as a microbial mats by Trower and Lowe (19). This sample is from the M1c member of the Mendon Formation, 250 m south of the Bruce's Hill locality. [B-E] Crinkly laminations from below S2 in the Bruce's Hill section. Laminations alternate with silica-rich layers, and contain siliciclastic and carbonaceous detrital grains. The laminations are 2-25 µm in thickness and can be traced for several centimeters, although especially the thicker laminations are composed of stacks of shorter and finer laminations. The laminations are strongly non-isopachous; they show bifurcation and anastomosis. Individual tufts and domes can reach up to 250 µm in topography. Detrital grains are draped and bound by organic matter, rarely showing any grain-to-grain contact with other detrital particles. They are oriented parallel to subparallel to bedding. The presence of detrital particles within the layers of crinkly laminations together with the close physical association with sandstone layers (e.g., bottom of image [B]) indicate a periodically current-stressed environment. [F+G] Relatively smooth carbonaceous laminations from in the Bruce's Hill and Umbaumba sections. These examples show sequences [F] abatement and [G] condensation (Noffke et al., accepted). These low-relief laminations show similar characteristics to thin laminations illustrated in Fig. S4. These layers are associated with few siliciclastic particles, suggesting a rather quiet-water environment.

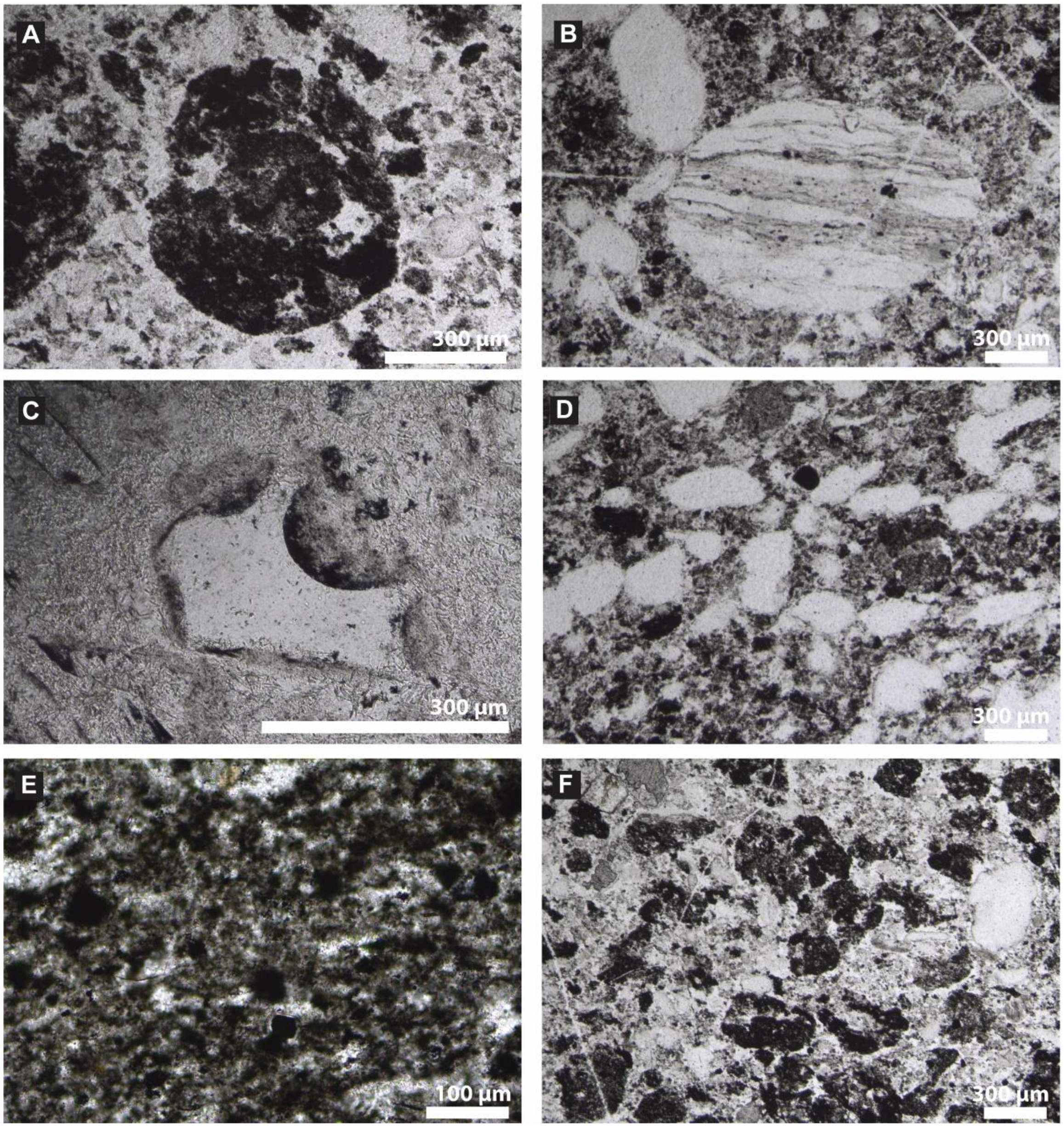

Fig. S6. Photomicrographs of carbonacoues particles from the Umbaumba and Bruce's Hill sections. [A] Complex carbonaceous grain in a matrix of simple carbonaceous grains and pure chert clasts. Simple carbonaceous grains are the most common type and occur in most carbonaceous chert bands. They are generally ≤150 µm in size, exhibit irregular shapes with ragged edges and no internal structuring. These particles range from fairly equant in shape to shapes with a high aspect ratio with increasing flattening due to compaction. Complex carbonaceous grains are sand-sized particles composed of two or more clots of unstructured organic matter bound within a silica matrix. They are generally larger (≥ 200 µm) than simple carbonaceous grains. They appear to be absent in strongly reworked detrital layers. [B] Intraclast of reworked laminated chert. These clasts are typically elongate and ≥ 500 µm in the long axis. [C] Coatings on detrital particles, such as seen here on a silicified pumice fragment, are up to 30 µm-thick and composed of fine carbonaceous matter. [D] Detrital layer composed of pure chert grains, simple carbonaceous particles, and less abudant siliciclastic debris. [E] Simple carbonaceous particles and fine siliciclastic debris from the top of the fallback layer in the Umbaumba section. [F]

Complex and simple carbonaceous grains together with pure chert grains from a detrital layer below S2 in the Umbaumba section.

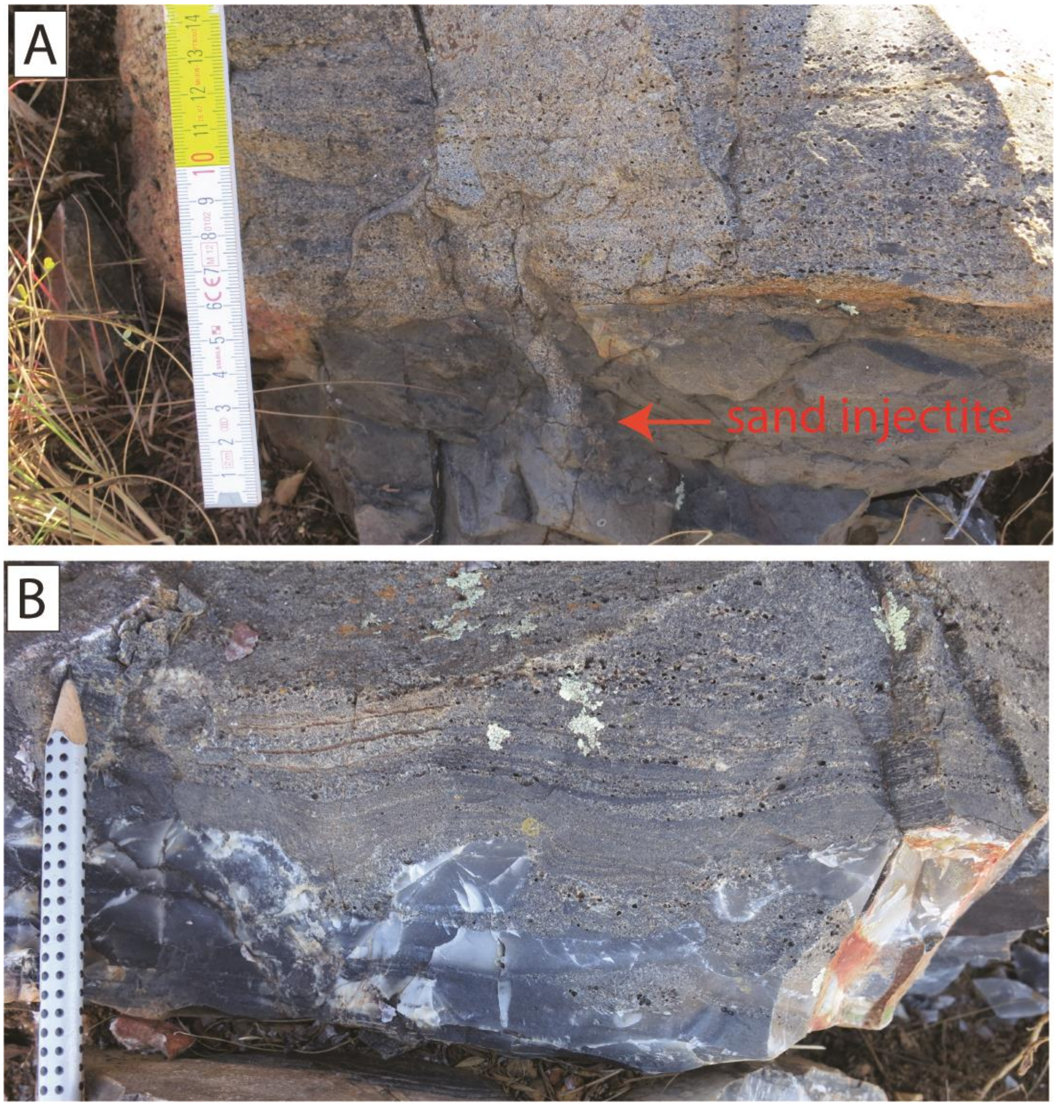

Fig. S7. Sedimentary structures in the Bruce's Hill section. [A] Sand injectite from the second spherule-bearing sandstone into the underlying fallback layer in the Bruce's Hill section at ~3.1 m in the stratigraphic section (Fig. 2). [B] Sandstone and chert above the third sperhule-bearing sandstone in the Bruce's Hill section at ~6 m in the stratigraphic section (Fig. 2). Sand erodes into the underlying chert, which shows evidence for ductile deformation. In other places, sand was injected into the tranlucent chert beds. This indicates that the translucent chert was not lithified at the time of sand deposition.

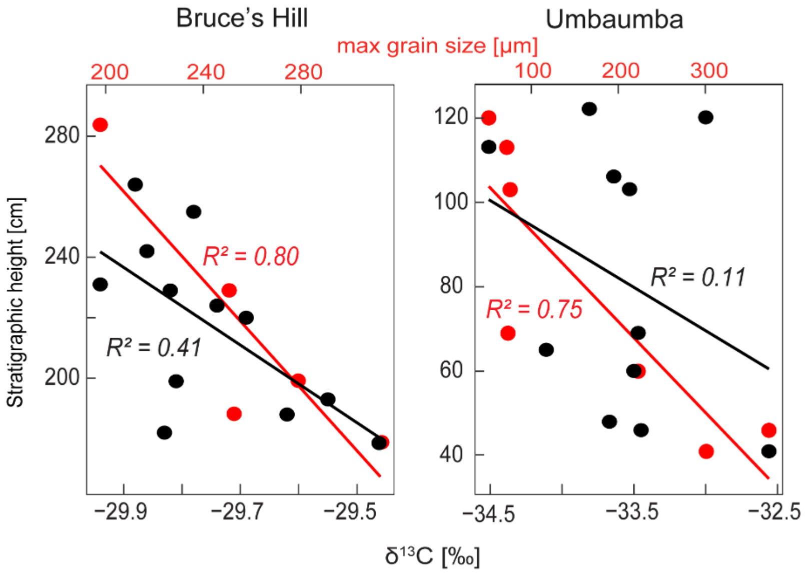

Fig. S8. Organic matter carbon isotopes (black circles) and grain size measurements (red circles) of the fallback layers above S2. $\delta^{13}C_{org}$ measurements show a moderate (Bruce's Hill) to weak (Umbaumba) trend of decreasing $\delta^{13}C_{org}$ with stratigraphic height. This trend correlates with a decrease in grain size.

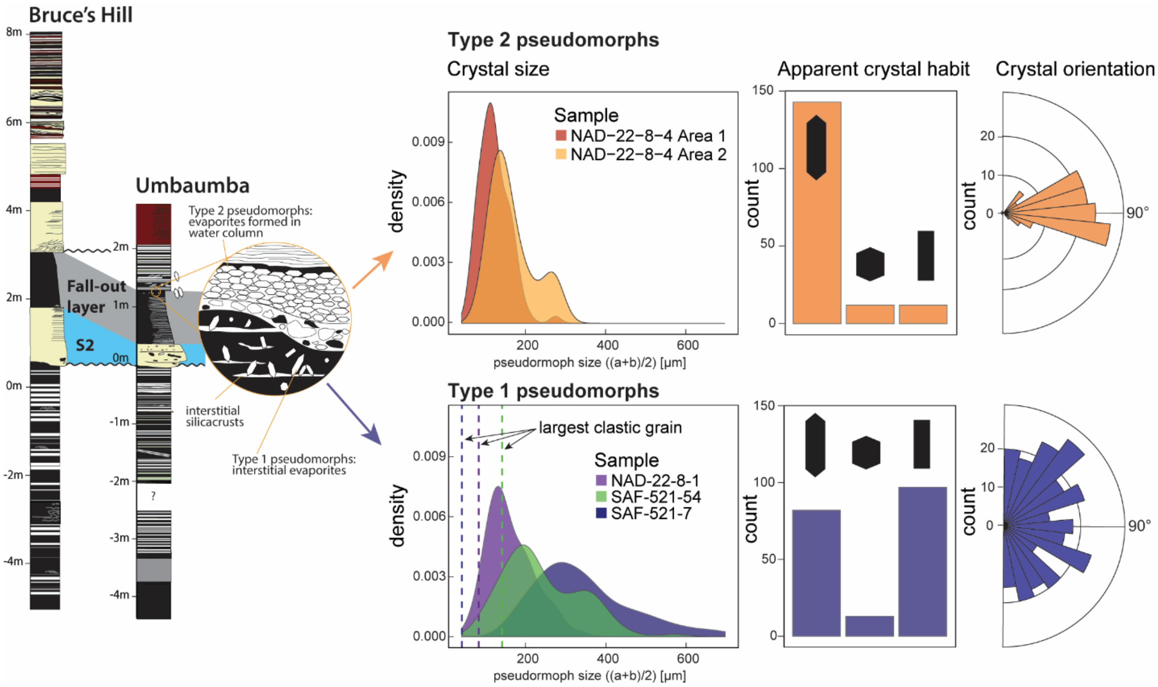

Fig. S9. Analyses of pseudomorphs in the Umbaumba section. Type 1 pseudomorphs are from within the upper fallback layer and Type 2 from above the fallback layer. Data was collected from thin sections. While this data from 2D images is informative, 3D imaging of pseudomorphs will be necessary to identify their true habit, and to assess variations in habits between the two types of pseudomorphs.

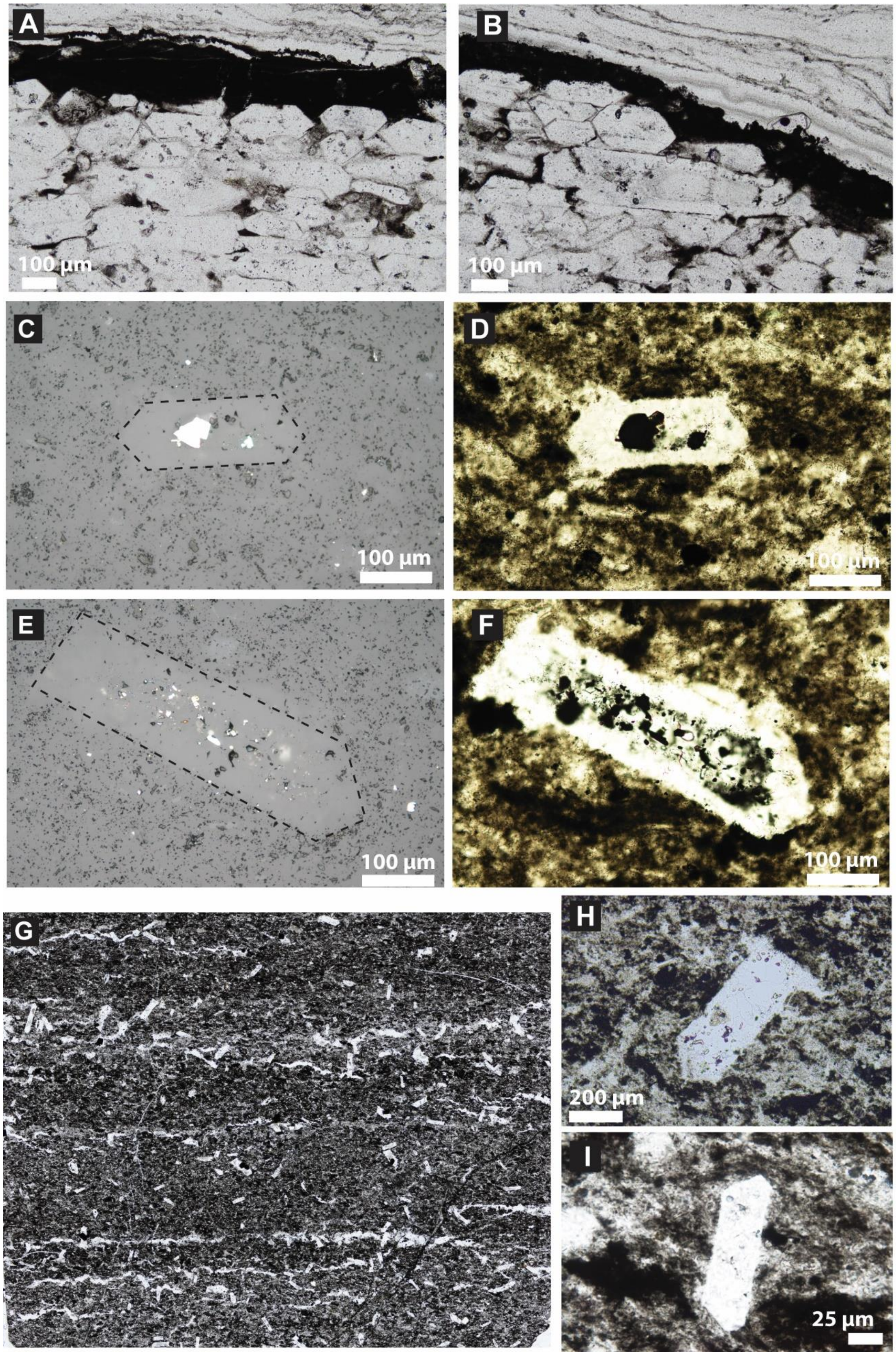
A
100 µm
B
100 µm
C
100 µm
D
100 µm
E
100 µm
F
100 µm
G
H
200 µm
I
25 µm

Fig. S10. Pseudomorph photomicrographs from the Umbaumba section. [A] and [B] show detailed images of Type 2 pseudomorphs above the fallback layer. Pseudomorphs show pseudohexagonal crystal habits and little to no evidence for abrasion. This layer is overlain by a pure carbonaceous layer, followed by chert with abundant fine carbonaceous laminations. Reflected light [C,E] and transmitted light images [D, F] of Type 1 pseudomorphs. Their inner edges are lined with finely crystalline quartz and their cores filled with coarsely crystalline quartz and, in some samples, pyrite. [G] Overview photo of randomly oriented pseudomorphs from the fallback layer in the Umbaumba section. [H] and [I] show Type 1 pseudomorphs from the fallback layer.

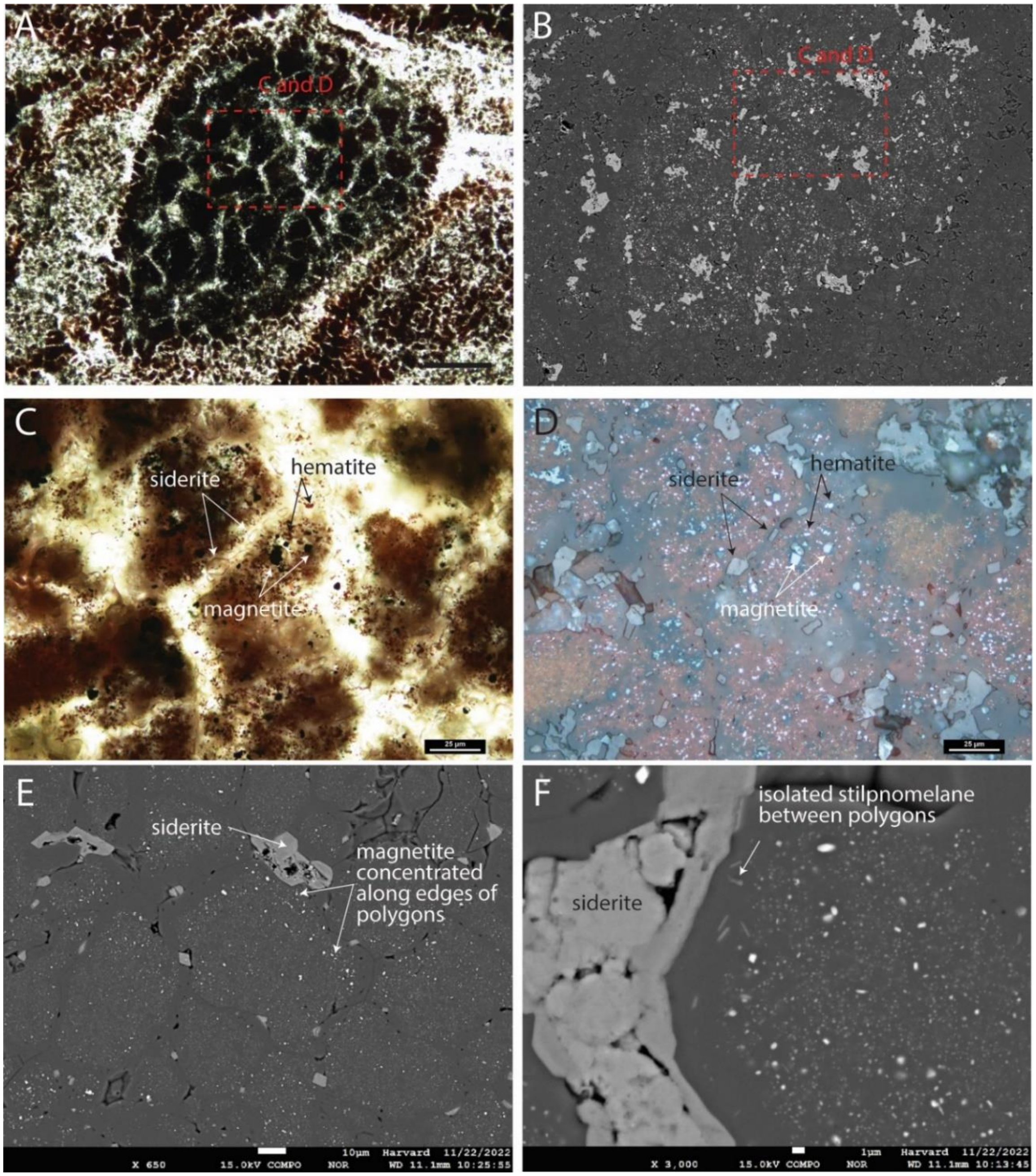

Fig. S11. Images of Fe grains in the Bruce's Hill section. Relatively uncompacted iron grain in [A] transmitted light and [B] SEM compositional map. [C] and [D] zoom into iron grain showing well-developed polygonal structures filled with micron-sized hematite and magnetite. The polygons are an early diagenetic feature that formed through desiccation of the sediment. Interstitial areas between polygons are filled with silica and commonly contain siderite. [E] Polygons composed of abundant sub-micron hematite. Magnetite tends to occur along the edges of the polygons, indicating a secondary origin. [F] Polygon with sub-micron hematite and some isolated magnetite. Stipnomelane and siderite occur between polygons, also suggesting an early diagenetic origin.

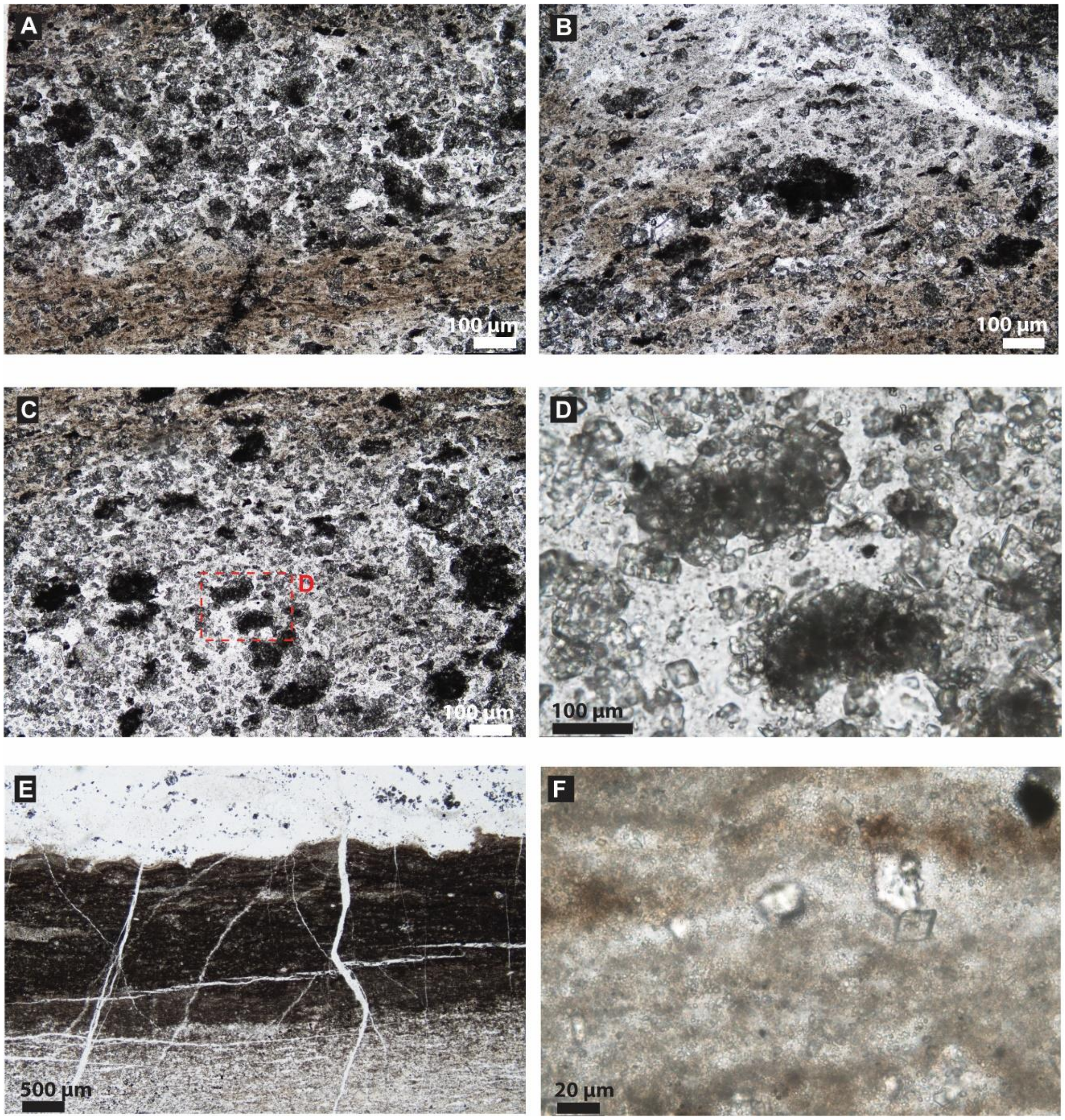

Fig. S12. Photomicrographs of siderite-rich layers. [A-D] Dark clots of carbonaceous matter and small siderite rhombs. Images are from siderite-rich layers above the S2 spherule bed in the Umbaumba section. [E-F] Siderite-rich layer from above the S2 spherule bed in the Bruce's Hill section. [E] Siderite-rich layer overlain by white chert band composed of silica granules (19). Siderites are generally <4 mircon and round(ish), or 20-200 micron and euhedral. Much of the dark, cloudy material in [F] represents organic matter; this layer has a TOC of 0.34 wt.%.

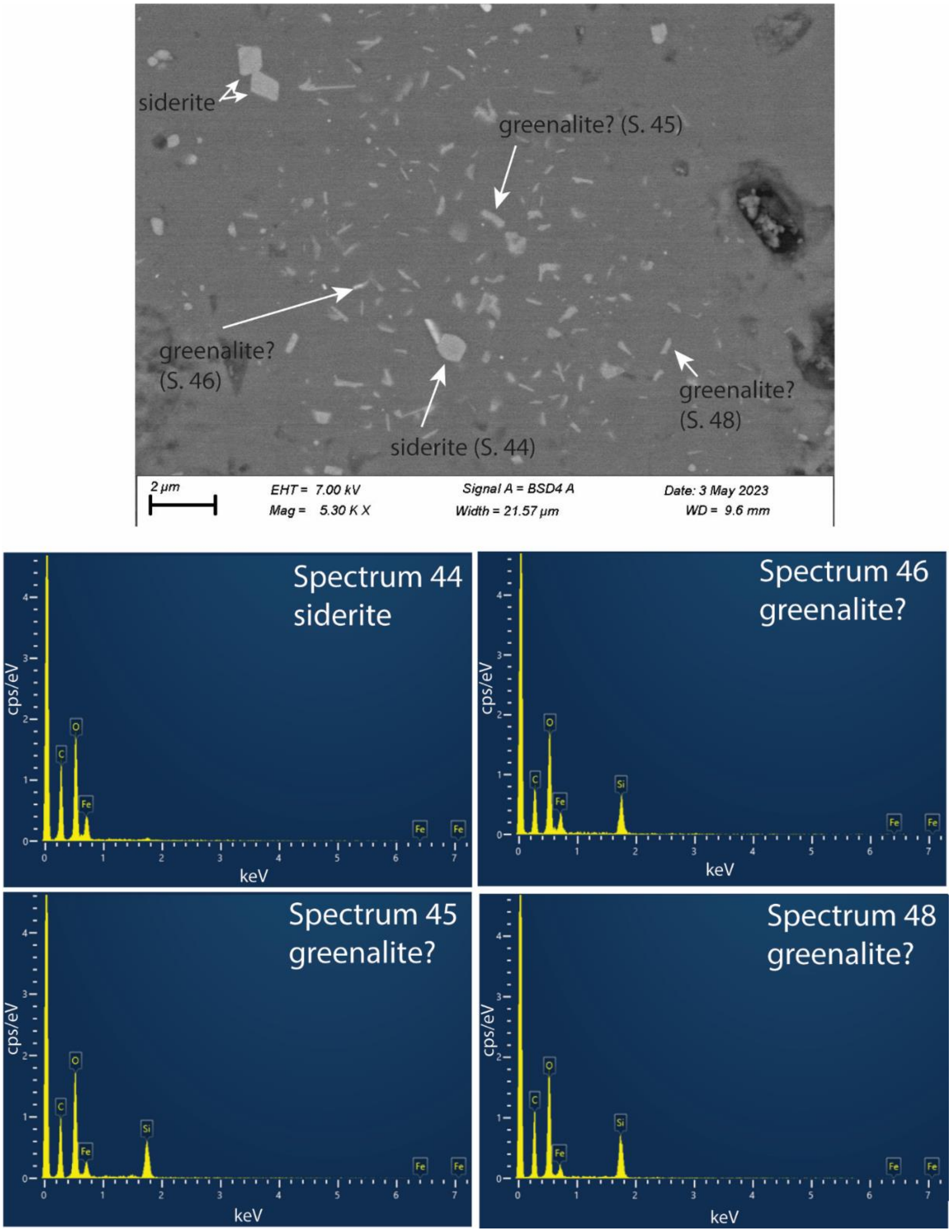

Fig. S13. Microprobe image and analyses of possible greenalite. SEM image of sample BH+580 from Bruce's Hill. Sample is composed of abundant ≤3 µm, round siderite with occasional stilpnomelane and possible greenalite. Image shows ~1 micron siderite together with abundant possible greenalite in a matrix of chert. EDS analyses are of siderite (Spectrum 44) and possible greenalite (Spectra 44, 45, 48). The possible greenalites are elongate grains ≤ 1µm in length. The random orientation suggests a diagenetic origin. The possible greenalites contain exclusively Fe and Si, albeit some Si may be scatter from the surrounding chert matrix. For full identification, transmission electron microscopy (TEM) analyses of an extraction foil will be necessary. All analyses contain carbon from the carbon coat.

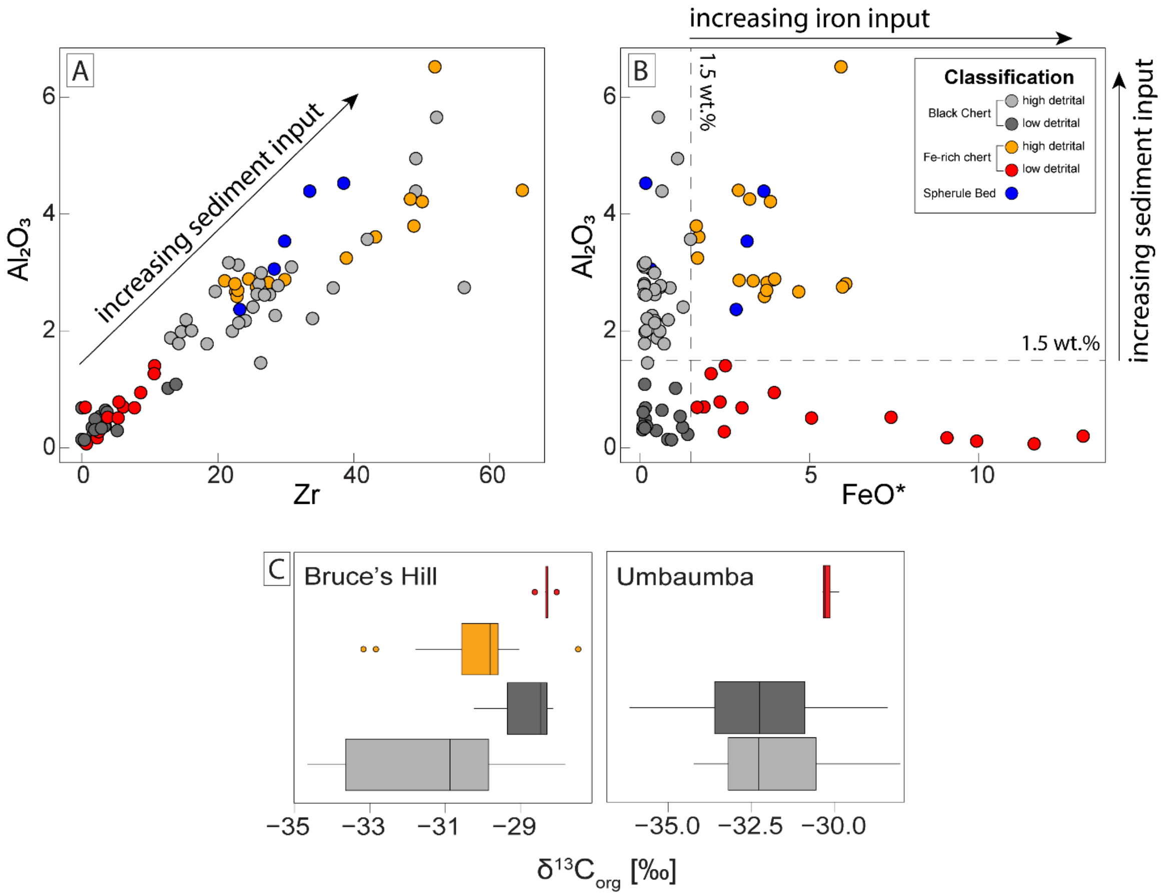

Fig. S14. Geochemical classification of samples. [A] Al_2O_3 correlates with Zr (and Cr, Th, Ti) and thus indicates sediment input. [B] Classification of geochemical data based on Al_2O_3 and FeO* content. A boundary of 1.5 wt.% Al_2O_3 and FeO* was chosen arbitrarily. [C] $\delta^{13}C_{org}$ data of each section colored by geochemical classification. Fe-rich cherts with low detrital input show heavier and more homogenous signatures. See Figure 6 for plot of $\delta^{13}C_{org}$ data vs stratigraphic height colored by geochemical classification.

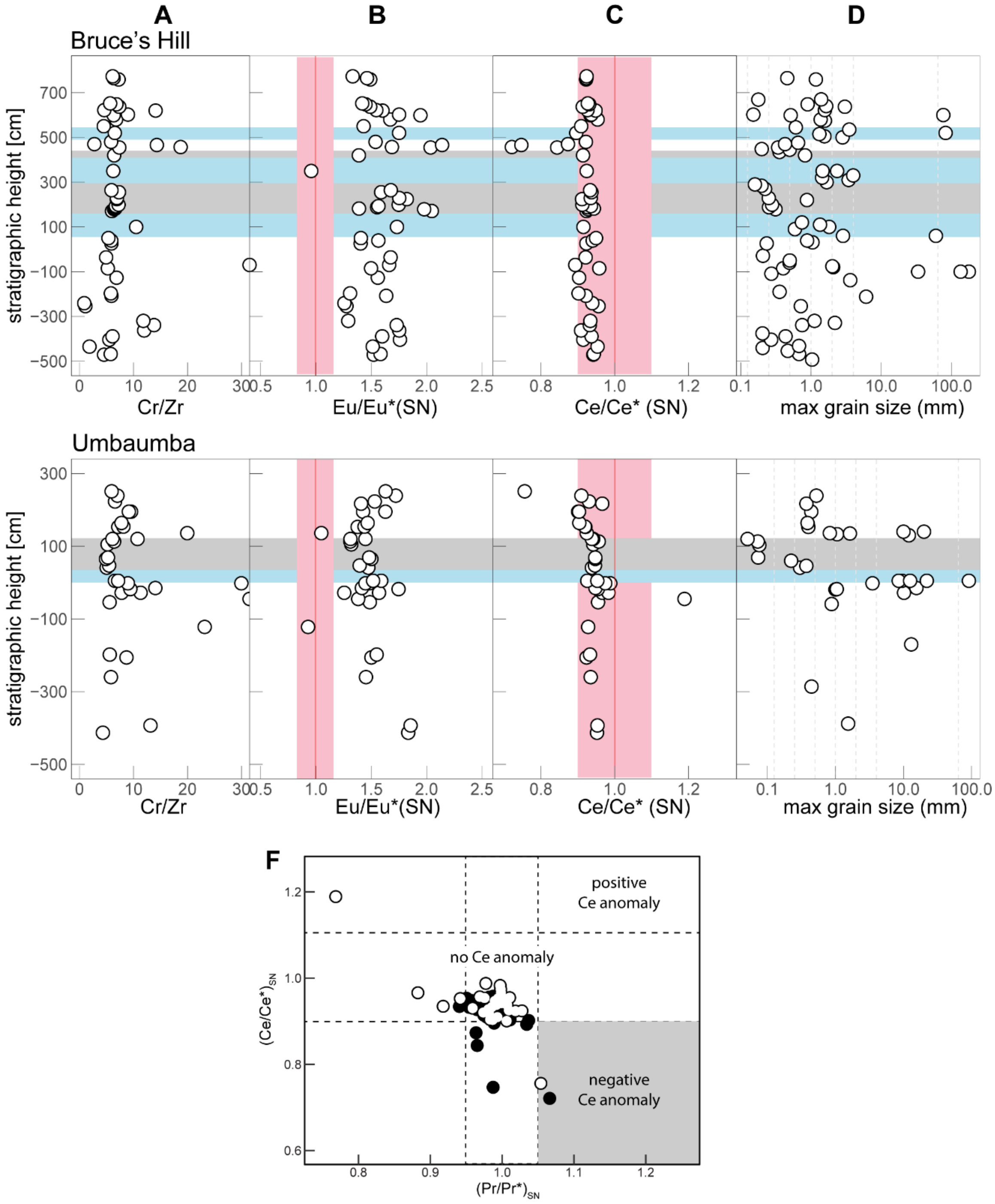

Fig. S15. Additional geochemical and grain size plots. [A] Cr/Zr, [B] PAAS-normalized Eu/Eu*, [C] normalized Ce/Ce*, [D] maximum grain size vs stratigraphic height. Red box in [B] and [C] reflects range that would indicate no anomaly. [F] $(Ce/Ce^*)_{SN}$ vs $(Pr/Pr^*)_{SN}$ data from Umbaumba (white) and Bruce's Hill (black) showing the range for true Ce anomalies (gray box).

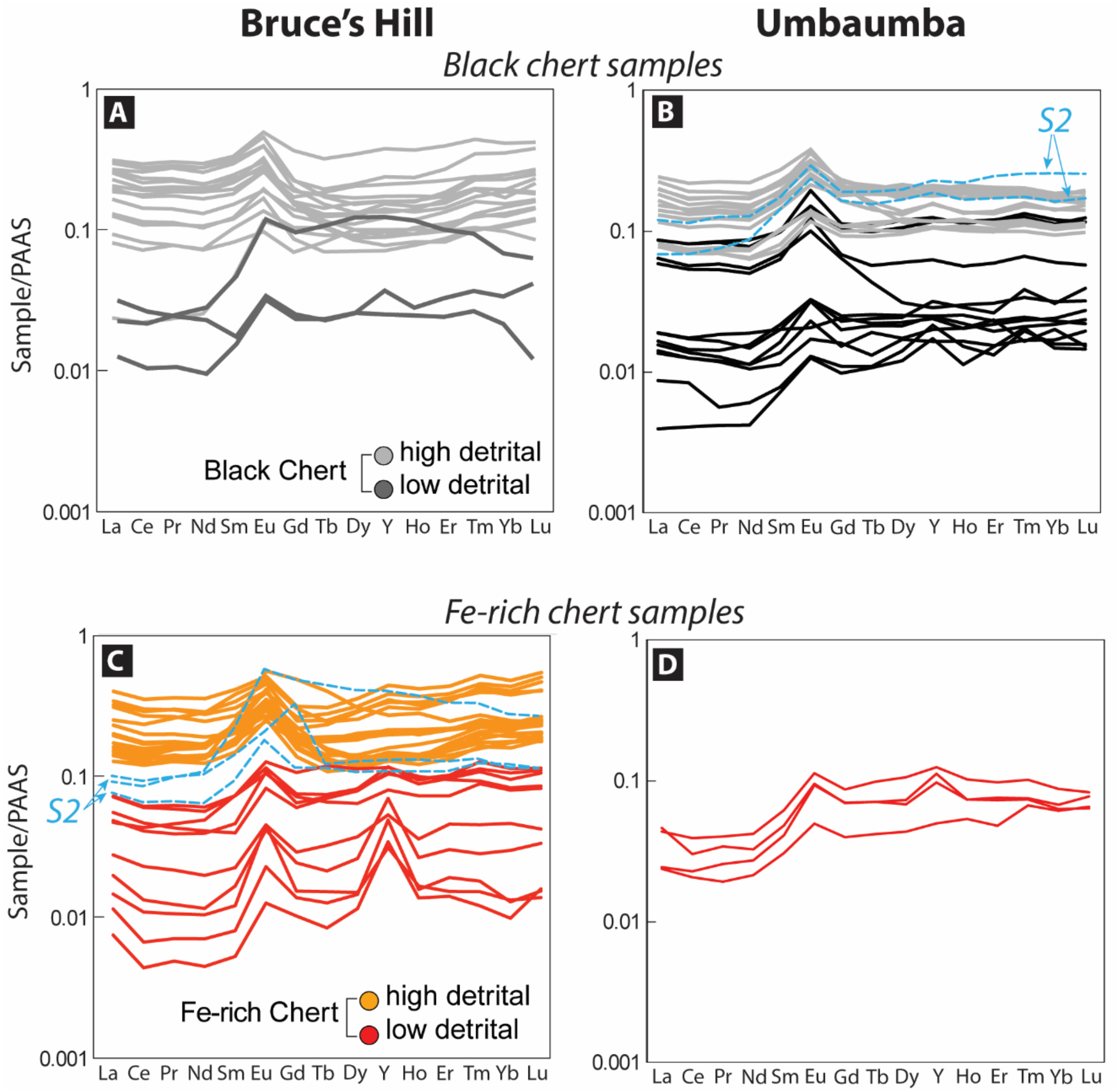

Fig. S16. REE+Y line graphs normalized to PAAS (22) **for the Bruce's Hill [A, C] and Umbaumba Sections [B, D]**. We use the same classification as shown in Figs. S10 and 6 based on Al_2O_3 and FeO*. [A + B] High and low detrital black chert samples. [C + D] High and low detrital Fe-rich chert samples.

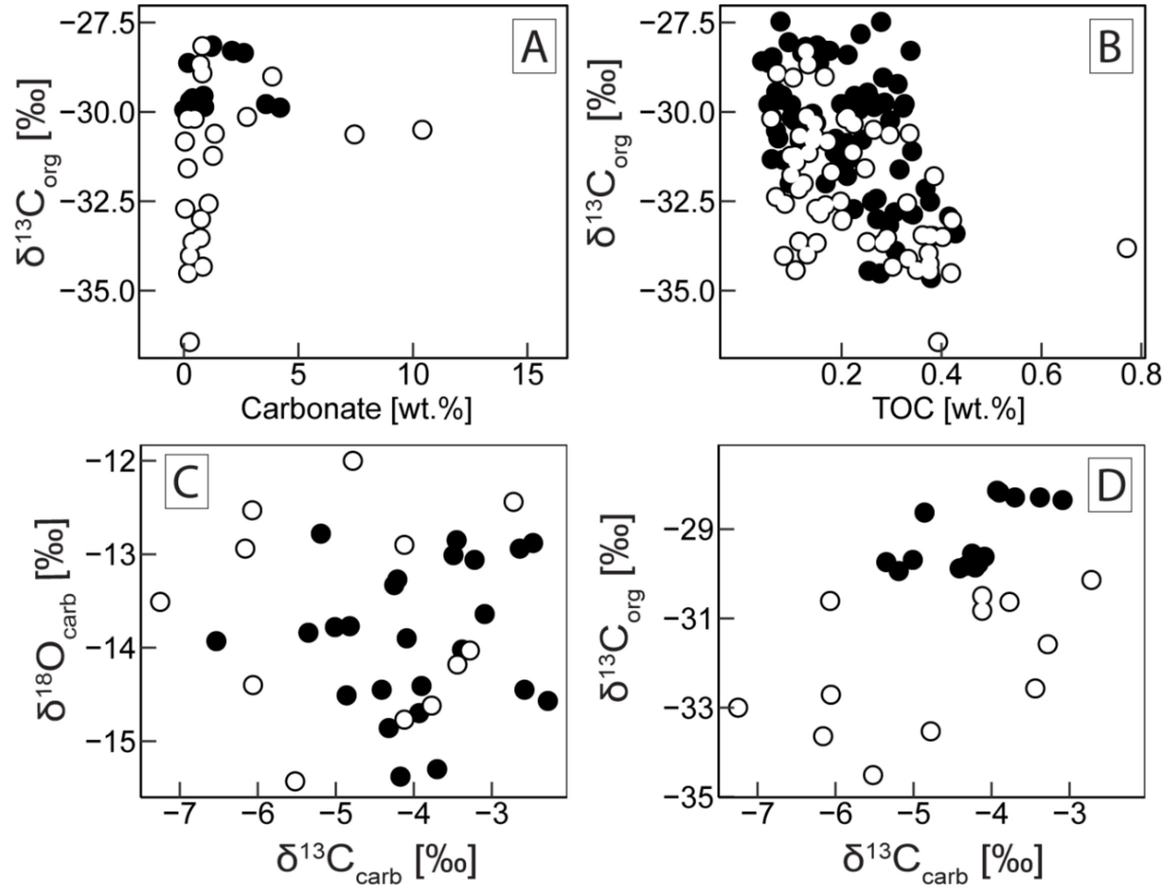

Fig. S17. Additional carbon isotope diagrams. [A] $\delta^{13}C_{org}$ [‰] vs carbonate [wt.%]. [B] $\delta^{13}C_{org}$ [‰] vs total organic carbon (TOC) [wt.%]. [C] $\delta^{18}O_{carb}$ [‰] vs $\delta^{13}C_{carb}$ [‰]. [D] $\delta^{13}C_{org}$ [‰] vs $\delta^{13}C_{carb}$ [‰]. Black circles are from Bruce's Hill and open circles are from Umbaumba.

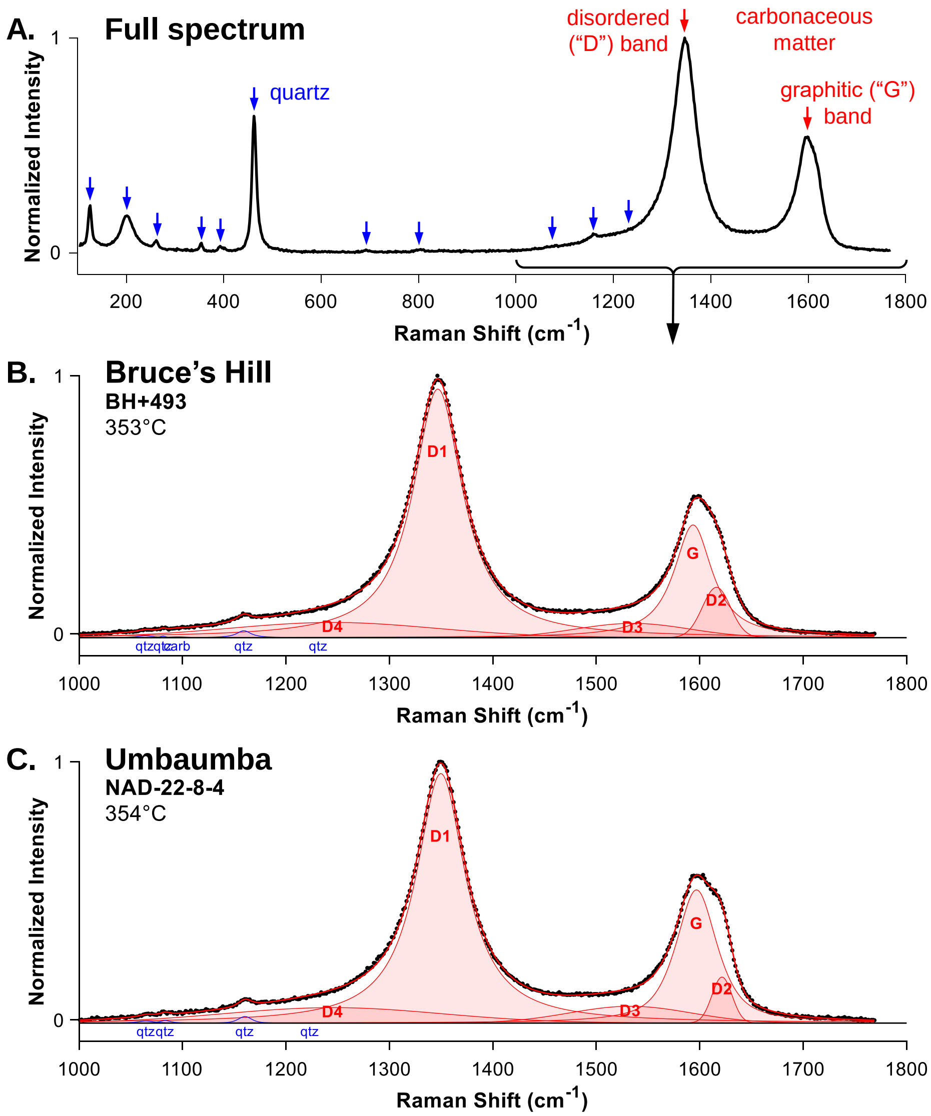

Fig. S18. Summary of Raman identification and geothermometry of carbonaceous matter. [A] A representative Raman spectrum of carbonaceous chert, showing features due to quartz (blue) and carbonaceous matter (red). The ~1350 cm^{-1} disordered low-crystallinity carbon "D" band and ~1600 cm^{-1} ordered graphitic carbon "G" band are apparent. [B] Fitting of the carbonaceous features in the spectrum from panel A, from a sample in the Bruce's Hill section, using the method of Kouketsu et al. (4) for geothermometry. Five peaks (D1-D4 and G, red shaded curves) and any peaks due to quartz or carbonate minerals (blue shaded curves) are fit to the spectrum (black points are data, superimposed red curve is the fit result) The full-width-at-half-maximum of the D1 peak indicates a peak maturation temperature of ~353°C, with systematic uncertainty of ~30°C. [C] Geothermometry of a representative sample from the Umbaumba section, with similar peak maturation temperature ~354°C.

Dataset S1 (separate file). Whole rock major, trace, and rare earth element geochemical data from the Peter Hopper GeoAnalytical Laboratory at Washington State University.

Dataset S2 (separate file). Carbonaceous matter carbon isotope ($\delta^{13}C_{org}$) data collected at Stanford University.

Dataset S3 (separate file). Total organic carbon (TOC) data. TOC analyses were conducted by GeoMark Research LLC (https://www.geomarkresearch.com/).

Dataset S4 (separate file). Carbonate carbon isotope ($\delta^{13}C_{carb}$) data collected at ETH Zurich.

Dataset S5 (separate file). Master spreadsheet with all data combined.

SI References

1. B. Durand, *Kerogen: Insoluble organic matter from sedimentary rocks*, Editions Technip (1980).
2. J. H. Parker, D. W. Feldman, M. Ashkin, Raman Scattering by Silicon and Germanium. *Physical Review* **155**, 712 (1967).
3. B. Lafuente, R. T. Downs, H. Yang, N. Stone, The power of databases: The RRUFF project. *Highlights in Mineralogical Crystallography* 1–29 (2016). https://doi.org/10.1515/9783110417104-003/HTML.
4. Y. Kouketsu, *et al.*, A new approach to develop the Raman carbonaceous material geothermometer for low-grade metamorphism using peak width. *Island Arc* **23**, 33–50 (2014).
5. C. P. Marshall, A. O. Marshall, Hematite and carbonaceous materials in geological samples: A cautionary tale. *Spectrochim Acta A Mol Biomol Spectrosc* **80**, 133–137 (2011).
6. S. F. M. Breitenbach, S. M. Bernasconi, Carbon and oxygen isotope analysis of small carbonate samples (20 to 100 µg) with a GasBench II preparation device. *Rapid Communications in Mass Spectrometry* **25**, 1910–1914 (2011).
7. A. Fernandez, J. van Dijk, I. A. Müller, S. M. Bernasconi, Siderite acid fractionation factors for sealed and open vessel digestions at 70 °C and 100 °C. *Chem Geol* **444**, 180–186 (2016).
8. X. Xie, G. R. Byerly, R. E. Ferrell Jr., IIb trioctahedral chlorite from the Barberton greenstone belt: crystal structure and rock composition constraints with implications to geothermometry. *Contributions to Mineralogy and Petrology* **126**, 275–291 (1997).
9. M. M. Tice, B. C. Bostick, D. R. Lowe, Thermal history of the 3.5-3.2 Ga Onverwacht and Fig Tree Groups, Barberton greenstone belt, South Africa, inferred by Raman microspectroscopy of carbonaceous material. *Geology* **32**, 37–40 (2004).
10. M. A. van Zuilen, M. Chaussidon, C. Rollion-Bard, B. Marty, Carbonaceous cherts of the Barberton Greenstone Belt, South Africa: Isotopic, chemical and structural characteristics of individual microstructures. *Geochim Cosmochim Acta* **71**, 655–669 (2007).
11. M. M. Walsh, D. R. Lowe, "Modes of accumulation of carbonaceous matter in the early Archean: A petrographic and geochemical study of the carbonaceous cherts of the Swaziland Supergroup" in *Geologic Evolution of the Barberton Greenstone Belt, South Africa*, D. R. Lowe, G. R. Byerly, Eds. (Geological Society of America, 1999), pp. 115–132.
12. M. M. Tice, D. R. Lowe, The origin of carbonaceous matter in pre-3.0 Ga greenstone terrains: A review and new evidence from the 3.42 Ga Buck Reef Chert. *Earth Sci Rev* **76**, 259–300 (2006).
13. K. Hickman-Lewis, F. Westall, A southern African perspective on the co-evolution of early life and environments. *South African Journal of Geology* **124**, 225–252 (2021).
14. K. Hickman-Lewis, F. Westall, B. Cavalazzi, Diverse communities of Bacteria and Archaea flourished in Palaeoarchaean (3.5–3.3 Ga) microbial mats. *Palaeontology* **63**, 1007–1033 (2020).

15. K. Hickman-Lewis, B. Cavalazzi, F. Foucher, F. Westall, Most ancient evidence for life in the Barberton greenstone belt: Microbial mats and biofabrics of the ∼3.47 Ga Middle Marker horizon. *Precambrian Res* **312**, 45–67 (2018).
16. N. Noffke, K. A. Eriksson, R. M. Hazen, E. L. Simpson, A new window into Early Archean life: Microbial mats in Earth's oldest siliciclastic tidal deposits (3.2 Ga Moodies Group, South Africa). *Geology* (2006). https://doi.org/10.1130/G22246.1.
17. N. Noffke, The criteria for the biogeneicity of microbially induced sedimentary structures (MISS) in Archean and younger, sandy deposits. *Earth Sci Rev* **96**, 173–180 (2009).
18. G. Gerdes, "Structures left by modern microbial mats in their host sediment" in *Atlas of Microbial Mat Features Preserved within the Clastic Rock Record*, J. , Schieber, *et al.*, Eds. (Elsevier, 2009), pp. 5–38.
19. E. J. Trower, D. R. Lowe, Sedimentology of the ∼3.3 Ga upper Mendon Formation, Barberton Greenstone Belt, South Africa. *Precambrian Res* **281**, 473–494 (2016).
20. C. R. Anhaeusser, L. J. Robb, M. J. Viljoen, Notes on the provisional geological map of the Barberton greenstone belt and surrounding granitic terrane, eastern Transvaal and Swaziland (1:250 000 colour map). *Spec. Publ. - Geol. Soc. S. Afr.* 221–223 (1983).
21. D. R. Lowe, Byerly G.R., C. Heubeck, Geologic map of the Barberton Greenstone Belt. *Geological Society of America." Map and Chart Series* **103** (2012).
22. S. R. Taylor, S. M. McLennan, *The Continental Crust: its Composition and Evolution. An Examination of the Geochemical Record Preserved in Sedimentary Rocks.* (1985).
